# Smartphone-based digital phenotyping for dry eye toward P4 medicine: a crowdsourced cross-sectional study

**DOI:** 10.1038/s41746-021-00540-2

**Published:** 2021-12-20

**Authors:** Takenori Inomata, Masahiro Nakamura, Jaemyoung Sung, Akie Midorikawa-Inomata, Masao Iwagami, Kenta Fujio, Yasutsugu Akasaki, Yuichi Okumura, Keiichi Fujimoto, Atsuko Eguchi, Maria Miura, Ken Nagino, Hurramhon Shokirova, Jun Zhu, Mizu Kuwahara, Kunihiko Hirosawa, Reza Dana, Akira Murakami

**Affiliations:** 1 Juntendo University Graduate School of Medicine, Department of Ophthalmology, Tokyo, Japan; 2 Juntendo University Graduate School of Medicine, Department of Strategic Operating Room Management and Improvement, Tokyo, Japan; 3 Juntendo University Graduate School of Medicine, Department of Hospital Administration, Tokyo, Japan; 4 Juntendo University Graduate School of Medicine, Department of Digital Medicine, Tokyo, Japan; 5 Precision Health, Department of Engineering, Graduate School of Bioengineering, The University of Tokyo, Tokyo, Japan; 6 University of South Florida, Morsani College of Medicine, Tampa, FL USA; 7 Department of Health Services Research, Faculty of Medicine, University of Tsukuba, Ibaraki, Japan; 8 Massachusetts Eye and Ear Infirmary, Department of Ophthalmology, Harvard Medical School, Boston, MA USA

**Keywords:** Predictive markers, Corneal diseases, Eye manifestations

## Abstract

Multidimensional integrative data analysis of digital phenotyping is crucial for elucidating the pathologies of multifactorial and heterogeneous diseases, such as the dry eye (DE). This crowdsourced cross-sectional study explored a novel smartphone-based digital phenotyping strategy to stratify and visualize the heterogenous DE symptoms into distinct subgroups. Multidimensional integrative data were collected from 3,593 participants between November 2016 and September 2019. Dimension reduction via Uniform Manifold Approximation and Projection stratified the collected data into seven clusters of symptomatic DE. Symptom profiles and risk factors in each cluster were identified by hierarchical heatmaps and multivariate logistic regressions. Stratified DE subgroups were visualized by chord diagrams, co-occurrence networks, and Circos plot analyses to improve interpretability. Maximum blink interval was reduced in clusters 1, 2, and 5 compared to non-symptomatic DE. Clusters 1 and 5 had severe DE symptoms. A data-driven multidimensional analysis with digital phenotyping may establish predictive, preventive, personalized, and participatory medicine.

## Introduction

Dry eye (DE) is a multifactorial ocular surface disorder^1,2^ causing impaired visual function, ocular discomfort, decreased quality of life, and reduced work productivity^3–6^. Its current worldwide prevalence is 5%–50%, which is expected to increase due to an aging society and increased digital work^7–9^. DE is characterized by a disruption in the tear-film homeostasis, including tear-film instability, hyperosmolarity, ocular inflammation, and subsequent damage of the ocular surface^1^. However, current treatment focuses on symptomatic relief *ex post facto*, with no convincing preventative or curative therapies^6^. Additionally, due to its wide heterogeneity and diverse symptom profiles, including photophobia, eyestrain, and reduced visual acuity^1^, symptoms are often overlooked as non-specific complaints, hindering optimal treatment selection for varying presentations. Therefore, predictive, preventive, personalized, and participatory (P4) medicine^10–12^ may facilitate the prevention of the onset and progression of DE via management optimization^12^. Subjective symptom measurement is the diagnostic standard in DE^2,6^, via validated questionnaires with a cutoff score^13^. However, this approach for DE diagnosis^13–15^ ignores various presentations and etiologies that could optimize treatment, which is further complicated by its multifactorial nature that includes environmental, host contributory, and lifestyle factors^7,9,16,17^. Therefore, monitoring individual subjective symptoms and contributory factors to elucidate and stratify the heterogeneous disease phenotype is needed for optimizing personalized treatments and managing personal risk factors^10,12,18^.

Mobile health (mHealth) refers to both medical and medical-care-supporting activities performed using smartphones^19^. The digital phenotype was first defined in 2016 as the “moment-by-moment quantification of the individual-level human phenotype in situ using data from personal digital devices,” which encompasses all data generated through an individual’s interaction with digital technologies. This includes, but is not limited to the data collected through embedded global positioning system (GPS), accelerometers, and photoplethysmography (pulse oximeters) in modern devices which allow instantaneous in situ measurements of human phenotypes^20^. Measurements collected through modern smart device-embedded sensors, along with patient-reported outcomes (PRO), are potential clinically relevant digital phenotypes^21^. mHealth’s novel features of collecting individual subjective symptoms, lifestyle data, and electronic PROs may prove useful as routinely collected digital phenotypes^22^.

**Fig. 1:**
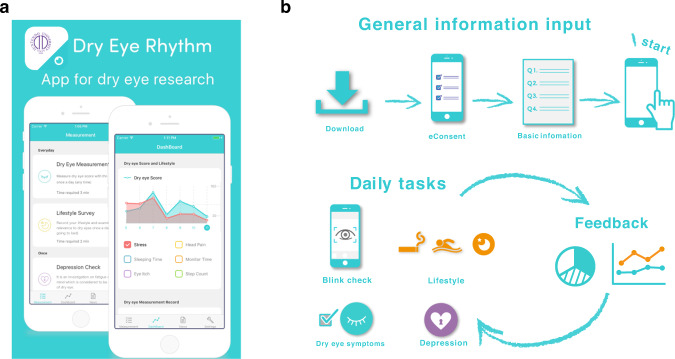
Screenshot and the description of user experience of DryEyeRhythm. (**a**) Screenshots of DryEyeRhythm (**b**) Description of the user experience of DryEyeRhythm. The copyright permission of the logo was obtained from Juntendo University, Tokyo, Japan.

We used “DryEyeRhythm (Fig. 1a),”—a free in-house application for DE—to longitudinally observe DE symptoms, related behaviors, and lifestyles, highlighting mHealth as an essential platform for digital phenotype data by identifying individual risk factors and DE characteristics (Fig. 1b)^9,17,23,24^. mHealth elucidates DE’s diversity and heterogeneity by stratifying multidimensional integrated data on heterogeneous symptoms, individual lifestyle factors, and biosensor data. mHealth, with the advancements made in mobile smart devices, allows for a non-intrusive, real-time, continuous collection of lifestyle data, and individual digital information which remains a challenge in traditional research methodologies^17^. Additionally, its capability for the comprehensive data collection on each individual, as well as subsequent personalized medical advice for the patient and public, have great implications in actuating all four pillars of P4 medicine^10,12^. We conducted a large-scale crowdsourced cross-sectional study using our DryEyeRhythm app to develop a robust method to stratify and visualize individual, heterogenetic subjective DE symptoms. Through advancing digital phenotyping for highly heterogeneous and diverse diseases, we attempt to lay a foundation for future P4 medicine^10,25,26^ in DE management by establishing an individual digital phenotyping protocol.

## Results

### Application description and study enrollment

**Fig. 2:**
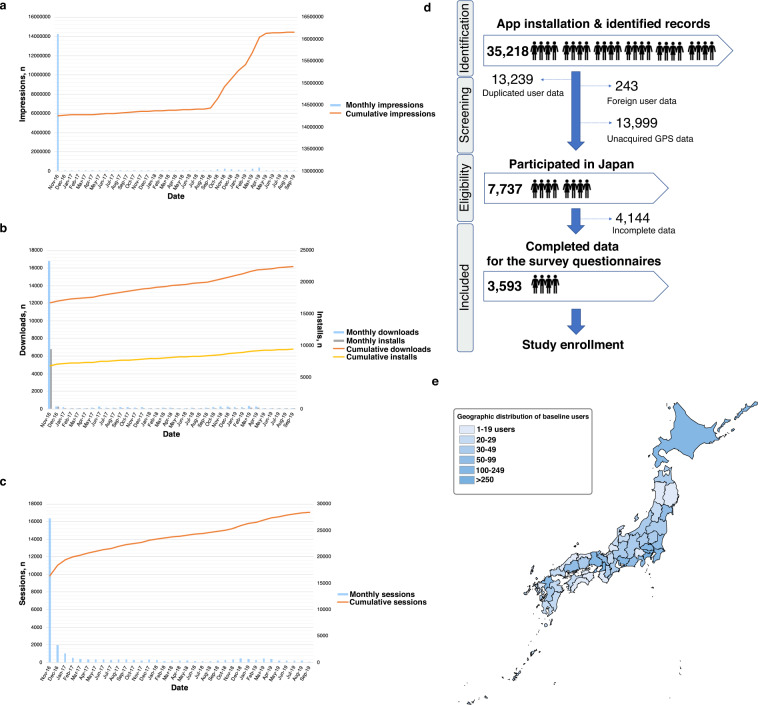
Study cohort description. Specific key performance indicators for DryEyeRhythm are shown in (**a**) impression times, (**b**) download times, and (**c**) session times. (**d**) Enrollment process of this study. (**e**) Geographic distribution of included participants (*n* = 3,593).

The DryEyeRhythm app was used to conduct the study. DryEyeRhythm’s key performance indicators between November 2, 2016, and September 30, 2019, are shown in Fig. 2(a–c). The total impression number was 16,161,564 times during this period (Fig. 2a). DryEyeRhythm was downloaded 22,810 times (Fig. 2b), with 28,408 total sessions (Fig. 2c). Study enrolment is presented in Fig. 2d; 35,218 records were identified in our crowd database, and 3,593 individuals completed the survey and were included. Supplementary Table 1 shows the sensitivity analysis between the included and excluded participants. Figure 2e shows the geographic distribution of the participants in Japan.

### Participant characteristics

Participants were DryEyeRhythm app users in Japan. Participant demographics are shown in Supplementary Table 2 (median age: 27 years; women: 59.8%). Symptomatic DE was defined as a total score ≥13 in the Japanese version of the Ocular Surface Disease Index (J-OSDI)^15^, which identified 2,619 (72.9%) individuals. Individuals with symptomatic DE tended to be younger (symptomatic DE vs. non-symptomatic DE; 26 years vs. 30 years; *P* < 0.001), female (65% vs. 45.8%; *P* < 0.001), have hay fever (52.3% vs. 45.9%; *P* < 0.001), and have a past DE diagnosis (28.8% vs. 14.5%; *P* < 0.001), compared with patients with non-symptomatic DE.

### Symptom-based stratification

**Fig. 3:**
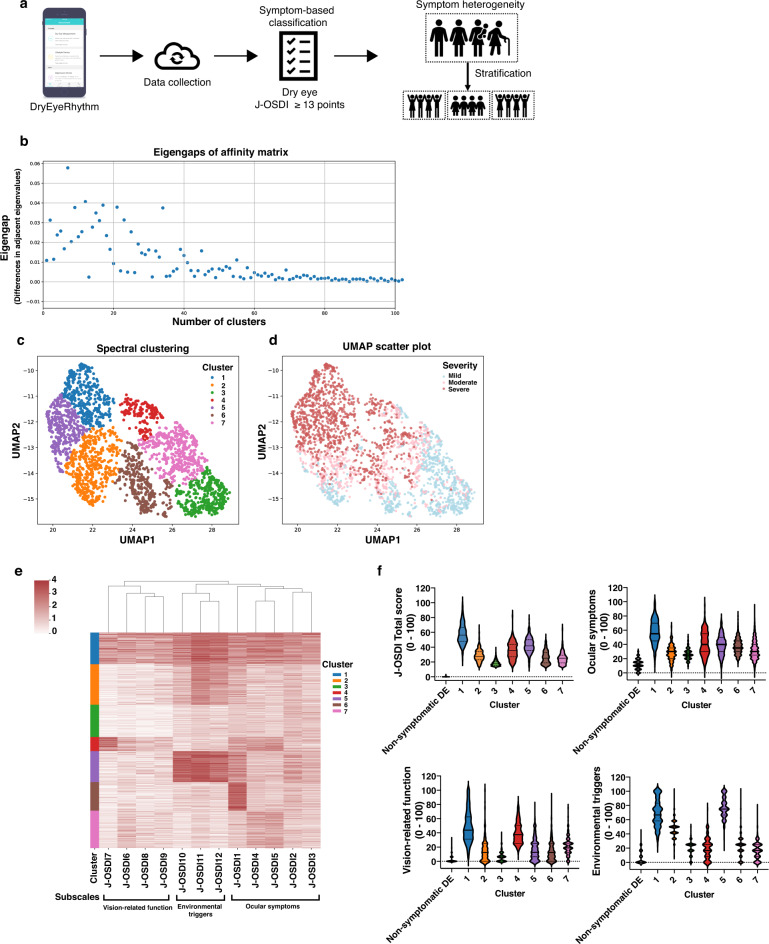
Stratification of subjective symptoms of DE. Heterogeneous and diverse subjective symptoms of DE were stratified using the 12 items of the J-OSDI. (**a**) An overview of the stratification process of heterogeneous and diverse subjective symptoms of DE using DryEyeRhythm. (**b**) Among symptomatic DE individuals, normalized maximum eigengap values were used to estimate the number of clusters during spectral clustering. Seven clusters were determined by eigengaps of the normalized affinity matrix. (**c**) Dimension reduction of individuals with symptomatic DE—via UMAP with spectral clustering identified by unsupervised clustering analysis *(n* = 2619 individuals collected by DryEyeRhythm)—depicted seven clusters when stratified for subjective symptoms based on the 12 items of the J-OSDI. (**d**) UMAP plots depicting subjective symptom severity of DE based on three subcategories of the J-OSDI. (**e**) Fraction of individuals within each cluster visualized on the left most panel, along with a corresponding heat map of subjective symptoms from individuals within the identified clusters. (**f**) Violin plots of total J-OSDI scores and each subscale, including ocular symptoms, vision-related function, and environmental triggers per cluster. Abbreviations: DE dry eye, J-OSDI Japanese version of Ocular Surface Disease Index, UMAP Uniform Manifold Approximation and Projection.

DE’s subjective symptoms were assessed using the 12-item J-OSDI questionnaire, and the participants were classified into two groups: non-symptomatic DE (J-OSDI total score <13) and symptomatic DE (J-OSDI total score ≥13). Our symptom-based stratification of the 12 J-OSDI items from 2,619 symptomatic individuals individually stratified DE’s heterogeneous symptoms (Fig. 3a). Seven clusters were determined using eigengaps of the normalized affinity matrix (Fig. 3b)^27^. Fig. 3c depicts a Uniform Manifold Approximation and Projection (UMAP) plot^28^ of those seven symptom-based stratified clusters using dimensional reduction analysis. Figure 3d depicts a UMAP plot clustered by DE severity based on the total J-OSDI score^15^. Fig. 3e shows a hierarchical heatmap illustrating individual DE profiles based on the stratified clusters with a dendrogram clustering the 12 J-OSDI items above the heatmap. Figure 3f shows a violin plot of total J-OSDI scores and each subscale, including ocular symptoms, vision-related function, and environmental triggers per cluster^14,15^. Cluster 1 showed the most severe symptoms in all the subscales, whereas cluster 5 showed the following severe symptoms with the higher score of the subscale of the environmental triggers.

Table 1 shows the participant demographics in each stratified cluster and the non-symptomatic DE (cluster 0); the median age was significantly younger in cluster 2 (24 years, *P* < 0.001) and cluster 5 (22 years, *P* < 0.001) compared with cluster 0 (30 years); cluster 4 (36 years, *P* < 0.001) showed significantly older median age compared with cluster 0. Asthenopia, mental fatigue, and body axis muscle stiffness and pain were mostly reported in cluster 1 (83.6%, 43.9%, and 68.6%, respectively). The highest scores for DE and depression, measured by J-OSDI and the Self-Rating Depression Scale (SDS), respectively, were from cluster 1, followed by cluster 5.

**Table 1:** Stratified participant characteristics by cluster.

	Non-symptomatic DE	Symptomatic DE						
	Cluster 0	Cluster 1	Cluster 2	Cluster 3	Cluster 4	Cluster 5	Cluster 6	Cluster 7
Demographics	*n* = 974 (27.1)	*n* = 385 (10.7)	*n* = 492 (13.7)	*n* = 393 (10.9)	*n* = 171 (4.8)	*n* = 376 (10.5)	*n* = 347 (9.7)	*n* = 455 (12.7)
Age (years), median [IQR]	30 [20–44]	27 [21–42]	24 [18–32]	28 [20–40]	36 [25–48]	22 [17–30]	27 [19–41]	32 [20–46]
Age (years), number (%)								
<20	214 (22.0)	82 (21.3)	153 (31.1)	90 (22.9)	13 (7.6)	148 (39.4)	90 (25.9)	106 (23.3)
20–30	263 (27.0)	138 (35.8)	190 (38.6)	125 (31.8)	49 (28.7)	129 (34.3)	108 (31.1)	100 (22.0)
30–40	190 (19.5)	57 (14.8)	71 (14.4)	75 (19.1)	32 (18.7)	57 (15.2)	59 (17.0)	71 (15.6)
40–50	174 (17.9)	59 (15.3)	50 (10.2)	62 (15.8)	41 (24.0)	31 (8.2)	55 (15.9)	92 (20.2)
50–60	98 (10.1)	38 (9.9)	20 (4.1)	31 (7.9)	29 (17.0)	9 (2.4)	26 (7.5)	61 (13.4)
≥60	35 (3.6)	11 (2.9)	8 (1.6)	10 (2.5)	7 (4.1)	2 (0.5)	9 (2.6)	25 (5.5)
Women, number (%)	446 (45.8)	263 (68.3)	355 (72.2)	241 (61.3)	94 (55.0)	298 (79.3)	208 (59.9)	242 (53.2)
Height (cm), median [IQR]	165 [158–172]	161 [156–168]	160 [156–167.5]	162 [157–168]	164 [157–172]	160 [155.5–164.5]	162 [157–169]	164 [157–171]
Body weight (kg), median [IQR]	60 [52–68]	56 [50–65]	53 [48–63]	56 [49–67]	58 [50–69]	54 [48–61]	57 [50–68]	59 [50–68]
Body mass index^a^, median [IQR]	21.8 [19.8–24.2]	21.5 [19.6–24.1]	20.7 [19.1–22.7]	21.5 [19.6–23.7]	22.0 [20.0–24.2]	20.9 [19.3–22.8]	21.5 [19.7–24.6]	21.6 [19.6–24.7]
Medical history								
Medicated hypertension, number (%)	46 (4.7)	14 (3.6)	12 (2.4)	14 (3.6)	14 (8.2)	5 (1.3)	19 (5.5)	28 (6.2)
Diabetes, number (%)	22 (2.3)	6 (1.6)	5 (1.0)	8 (2.0)	7 (4.1)	3 (0.8)	5 (1.4)	10 (2.2)
Systemic diseases, number (%)								
Blood disease	12 (1.2)	8 (2.1)	5 (1.0)	1 (0.3)	1 (0.6)	1 (0.3)	2 (0.6)	6 (1.3)
Brain disease	9 (0.9)	4 (1.0)	4 (0.8)	4 (1.0)	2 (1.2)	1 (0.3)	5 (1.4)	1 (0.2)
Collagen disease	5 (0.5)	7 (1.8)	4 (0.8)	1 (0.3)	1 (0.6)	6 (1.6)	3 (0.9)	5 (1.1)
Heart disease	16 (1.6)	9 (2.3)	7 (1.4)	2 (0.5)	6 (3.5)	9 (2.4)	6 (1.7)	15 (3.3)
Kidney disease	13 (1.3)	6 (1.6)	11 (2.2)	11 (2.8)	3 (1.8)	6 (1.6)	6 (1.7)	9 (2.0)
Liver disease	13 (1.3)	6 (1.6)	2 (0.4)	4 (1.0)	5 (2.9)	3 (0.8)	8 (2.3)	9 (2.0)
Malignant tumor	8 (0.8)	4 (1.0)	6 (1.2)	0 (0.0)	1 (0.6)	0 (0.0)	3 (0.9)	5 (1.1)
Respiratory disease	58 (6.0)	31 (8.1)	42 (8.5)	29 (7.4)	13 (7.6)	26 (6.9)	25 (7.2)	33 (7.3)
Hay fever, number (%)	447 (45.9)	212 (55.1)	255 (51.8)	194 (49.4)	82 (48.0)	212 (56.4)	187 (53.9)	228 (50.1)
Mental illness, number (%)								
Depression	29 (3.0)	31 (8.1)	21 (4.3)	15 (3.8)	15 (8.8)	15 (4.0)	24 (6.9)	17 (3.7)
Schizophrenia	9 (0.9)	5 (1.3)	2 (0.4)	4 (1.0)	2 (1.2)	4 (1.1)	1 (0.3)	4 (0.9)
Others	32 (3.3)	32 (8.3)	21 (4.3)	25 (6.4)	12 (7.0)	22 (5.9)	20 (5.8)	27 (5.9)
Past diagnosis of dry eye, number (%)	141 (14.5)	169 (43.9)	126 (25.6)	93 (23.7)	40 (23.4)	153 (40.7)	72 (20.8)	100 (22.0)
Ophthalmic surgery, number (%)								
Cataract surgery	10 (1.0)	2 (0.5)	1 (0.2)	2 (0.5)	1 (0.6)	0 (0.0)	1 (0.3)	4 (0.9)
LASIK	23 (2.4)	4 (1.0)	6 (1.2)	6 (1.5)	7 (4.1)	5 (1.3)	5 (1.4)	7 (1.5)
Others	24 (2.5)	20 (5.2)	11 (2.2)	19 (4.8)	4 (2.3)	6 (1.6)	10 (2.9)	11 (2.4)
Eye drops, yes	146 (15.0)	155 (40.3)	144 (29.3)	88 (22.4)	42 (24.6)	136 (36.2)	82 (23.6)	96 (21.1)
Lifestyle habits								
Coffee (cups per day), median [IQR]	1 [0–2]	1 [0–2]	0 [0–1]	0 [0–2]	1 [0–2]	0 [0–1]	0 [0–2]	1 [0–2]
Contact lens use, number (%)								
Negative	537 (55.1)	164 (42.6)	193 (39.2)	186 (47.3)	94 (55.0)	129 (34.3)	183 (52.7)	267 (58.7)
Current use	307 (31.5)	170 (44.2)	240 (48.8)	165 (42.0)	41 (24.0)	202 (53.7)	126 (36.3)	149 (32.8)
Past use	130 (13.4)	51 (13.3)	59 (12.0)	42 (10.7)	36 (21.1)	45 (12.0)	38 (11.0)	39 (8.6)
Screen exposure time (hours per day), median [IQR]	6 [4–10]	8 [4–10]	6 [4–10]	6 [4–10]	6 [4–10]	6 [4–10]	6 [4–10]	6 [4–10]
<4	196 (20.1)	62 (16.1)	82 (16.7)	65 (16.5)	33 (19.3)	65 (17.3)	53 (15.3)	81 (17.8)
4–8	532 (54.6)	184 (47.8)	259 (52.6)	196 (49.9)	86 (50.3)	201 (53.5)	200 (57.6)	232 (51.0)
>8	246 (25.3)	139 (36.1)	151 (30.7)	132 (33.6)	52 (30.4)	110 (29.3)	94 (27.1)	142 (31.2)
Periodic exercise, number (%)	645 (66.2)	227 (59.0)	331 (67.3)	245 (62.3)	85 (49.7)	235 (62.5)	224 (64.6)	300 (65.9)
Periodic exercise (hours per week), median [IQR]	1 [0–4]	1 [0–3]	1 [0–3]	1 [0–3]	0 [0–2]	1 [0–3]	1 [0–3]	1 [0–3]
Sleeping time (hours per day), median [IQR]	7 [6–8.5]	7 [5.6–8.5]	7 [6–8.5]	7 [6–8.75]	7 [6–8.6]	7.1 [6–8.4]	7.3 [6.1–8.8]	7 [6–8.3]
<6	286 (29.5)	135 (35.3)	135 (27.6)	115 (29.3)	47 (27.5)	106 (28.2)	85 (24.6)	111 (24.5)
6–9	516 (531.1)	179 (46.7)	265 (54.1)	193 (49.2)	92 (53.8)	210 (55.9)	197 (56.9)	272 (60.0)
>9	169 (17.4)	69 (18.0)	90 (18.4)	84 (21.4)	32 (18.7)	60 (16.0)	64 (18.5)	70 (15.5)
Smoking, number (%)	245 (25.2)	135 (35.1)	117 (23.8)	100 (25.5)	70 (40.9)	103 (27.4)	101 (29.1)	134 (29.5)
Water intake (100 mL per day), median [IQR]	8 [4–10]	8 [4–10]	7 [4–10]	8 [4–10]	8 [4–10]	8 [4–10]	8 [4–10]	8 [4–10]
Subjective symptoms								
Daily subjective symptoms								
Eye itching, (0, not at all to 10, very itchy), median [IQR]	0 [0–2]	4 [1–7]	2 [0–5]	2 [0–4]	2 [0–4]	3 [0–6]	2 [0–4]	2 [0–4]
Asthenopia, number (%)	395 (40.6)	322 (83.6)	301 (61.2)	224 (57.0)	130 (76.0)	261 (69.4)	218 (62.8)	317 (69.7)
Headache, (0, not at all to 10, very painful), median [IQR]	0 [0–2]	2 [0–5]	1 [0–3]	1 [0–3]	1 [0–4]	1 [0–5]	1 [0–4]	1 [0–3]
Mental fatigue, number (%)	172 (17.7)	169 (43.9)	142 (28.9)	102 (26.0)	54 (31.6)	54 (31.6)	116 (30.9)	108 (31.1)
Stiffness and pain of body axis muscles, number (%)	387 (39.7)	264 (68.6)	272 (55.3)	219 (55.7)	107 (62.6)	212 (56.4)	189 (54.5)	230 (50.6)
Stress, (0, not at all to 10, I feel very stressed), median [IQR]	3 [2–6]	6 [3–8]	5 [3–7]	5 [3–7]	5 [3–7]	5 [3–7]	5 [3–7]	5 [3–7]
J-OSDI, (0–100), median [IQR]								
J-OSDI total score	8.3 [4.5–10.4]	56.3 [47.9–66.7]	27.5 [22.9–34.1]	17.5 [15–20.8]	35.4 [27.1–43.8]	41.7 [35.4–50]	25 [18.8–32.5]	25 [18.8–29.2]
Ocular symptoms	10 [5–15]	55 [45–70]	30 [20–35]	25 [20–30]	40 [30–55]	40 [30–50]	35 [30–45]	30 [25–40]
Vision-related function	0 [0–6.3]	43.8 [31.3–62.5]	12.5 [0–25]	6.3 [0–8.3]	37.5 [25–50]	12.5 [6.3–25]	12.5 [0–25]	18.8 [12.5–25]
Environmental triggers	0 [0–16.7]	66.7 [58.3–83.3]	50 [41.7–50]	25 [16.7–25]	16.7 [8.3–25]	75 [66.7–85.4]	25 [16.7–25]	16.7 [8.3–25]
SDS								
SDS total score, (20–80), median [IQR]	39.5 [34–46]	48 [41–56]	45 [37.5–50]	44 [37–50]	46 [40–53]	47 [40–54]	45 [38–52]	44 [37–51]
Depressive symptoms score ≥40, number (%)	487 (50.0)	303 (78.7)	339 (68.9)	267 (67.9)	130 (76.0)	287 (76.3)	244 (70.3)	305 (67.0)

DE dry eye, IQR interquartile range, LASIK laser-assisted in situ keratomileusis, J-OSDI Japanese version of Ocular Surface Disease Index, SDS Zung Self-rating Depression Scale.

^a^Calculated as weight (in kilograms) divided by height (in meters) squared.

### Chord diagram and co-occurrence network analysis

**Fig. 4:**
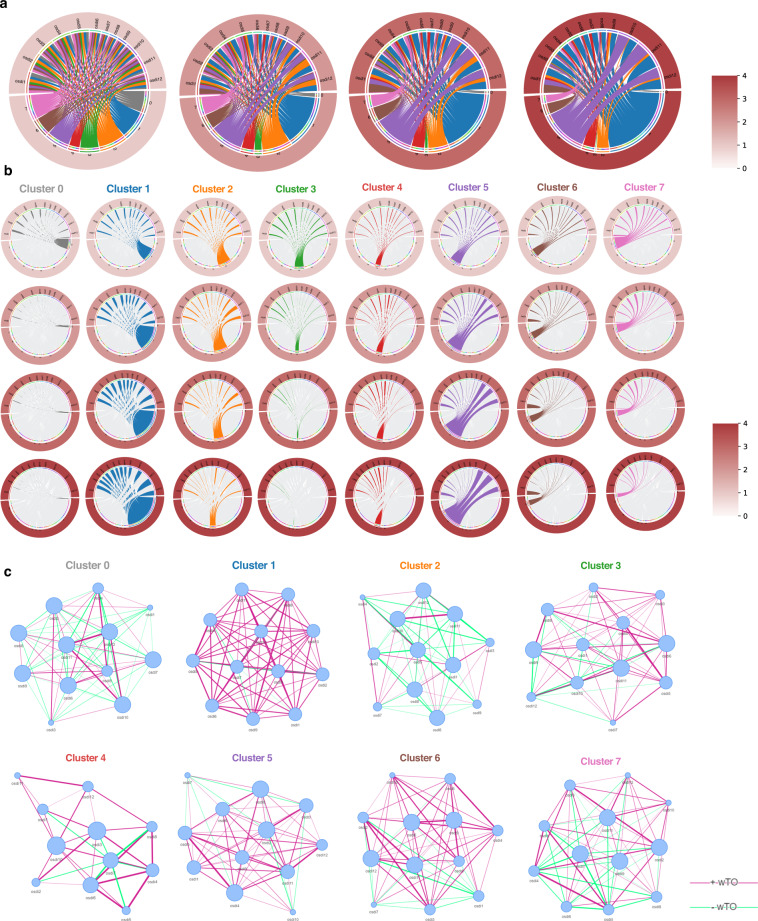
Chord diagram and co-occurrence network analysis. Chord diagrams visualizing the quantified interrelationships between each pair of stratified clusters, and the 12 items of the J-OSDI were placed around a circle. (**a**) Chord diagrams showing the J-OSDI-to-cluster interrelations for J-OSDI items with a score higher than 1 (left), 2 (middle left), 3 (middle right), and 4 (right). (**b**) The interrelationships of the stratified clusters and the 12 items of the J-OSDI are displayed separately for each cluster. (**c**) Co-occurrence network analysis displaying significant connection relationships between each of the 12 J-OSDI questions within each cluster. Co-occurrence network analyses were performed with wTO measure, which enables normalization of all shared correlations between a pair of parameters. In the co-occurrence network analysis representations, nodes (the 12 items of the J-OSDI) are represented as circles, and links between the nodes (inter-item correlations) are represented as lines. The size of each node is proportional to the frequency of a DE symptom that corresponds to a J-OSDI item. The width of the lines reflects the strength of the pairwise correlations on the 12 J-OSDI domains. To enable the comparison of edge strength across networks, a thicker edge identified across all networks implied a stronger association. Purple edges represent positive interconnections, whereas green edges represent negative interconnections. Abbreviations: J-OSDI Japanese version of Ocular Surface Disease Index, wTO weighted topological overlap, DE dry eye.

Figure 4 depicts a chord diagram, visualizing J-OSDI’s 12-item interrelationships per cluster. Figure 4a displays the chord diagrams, with threshold scores of 1–4 for each J-OSDI item, with the lowest score on the left. Proportions of clusters 1 and 5 and J-OSDI threshold scores were positively correlated, while the remaining clusters (0, 2, 3, 6, and 7) decreased. Figure 4b shows a per-cluster subdivision of the aforementioned chord diagram, displaying chord diagrams with threshold scores of 1–4 for each J-OSDI item with the lowest score on the top. In the leftmost panel, the proportional area of non-symptomatic DE (cluster 0) showed a decreasing trend as the threshold of the J-OSDI score increased. Areas of clusters 1 and 5 increased as the threshold of the J-OSDI score increased. Additionally, the chord diagram depicted an increased severity of all J-OSDI items for cluster 1. The severity of the three environmental J-OSDI items (items 10–12) was increased in cluster 5.

Co-occurrence network analyses provided the functions and interactions of the J-OSDI items at each cluster level (Fig. 4c). The resulting weighted topological overlap (wTO) and Pearson coefficient values are presented in Supplementary Table 3. Each J-OSDI item acted as a single node, and the inter-item pairwise partial associations were expressed as internodal lines of the diagram. Notably, non-symptomatic DE (cluster 0) displayed predominantly negative correlations with independent nodal activity. All nodes from cluster 1 displayed comparable signal intensities with predominantly positive internodal correlations. Cluster 4 displayed positive correlations with five nodes that correspond to ocular symptoms (items 4 and 5) and vision-related function in the J-OSDI (items 6–8). The proportion of nodes 7 and 10 was relatively small for cluster 5, while the remaining nodes demonstrated comparable signal intensities.

### Blinking biosensing

**Fig. 5:**
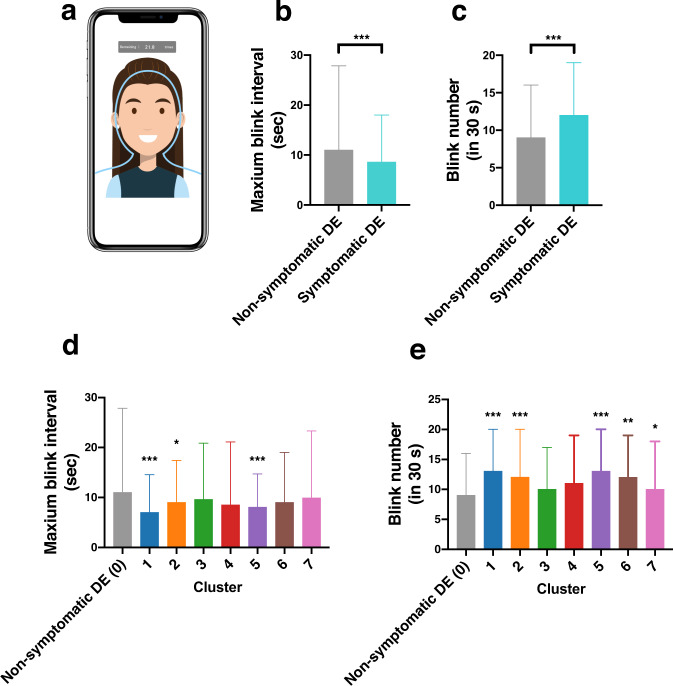
Characteristics of identified clusters. (**a**) The duration of the participants’ MBI and 30-s blinking frequency were recorded by DryEyeRhythm. (**b**) MBI in each cluster (Kruskal-Wallis test, n = 3593, *P = 0.016. ***P < 0.001). (**c**) Blink frequency (blink number) in each cluster (Kruskal-Wallis test, *n* = 3593, *P = 0.027. **P = 0.005, ***P < 0.001). Abbreviation: MBI maximum blink interval, DE dry eye.

Users’ own smartphone cameras and the CIFaceFeature were used to measure blinking. Figure 5a displays a representative screenshot for in-app blinking biosensing. The maximum blink interval (MBI)^29^ is a non-invasive screening method for DE. The MBI was significantly shortened in symptomatic DE (Fig. 5b, 8.6 s vs. 11.0 s, *P* < 0.001), while there was an opposite pattern in the 30-s blink frequency (12 times vs. 9 times, *P* < 0.001, in patients with symptomatic DE and non-symptomatic DE, respectively) (Fig. 5c). Compared with non-symptomatic DE, the MBI was significantly shorter in clusters 1, 2, and 5 (Fig. 5d). The 30-s blink frequency was significantly higher in clusters 1, 2, 5, and 7 (Fig. 5e).

### Risk factors for each cluster

**Fig. 6:**
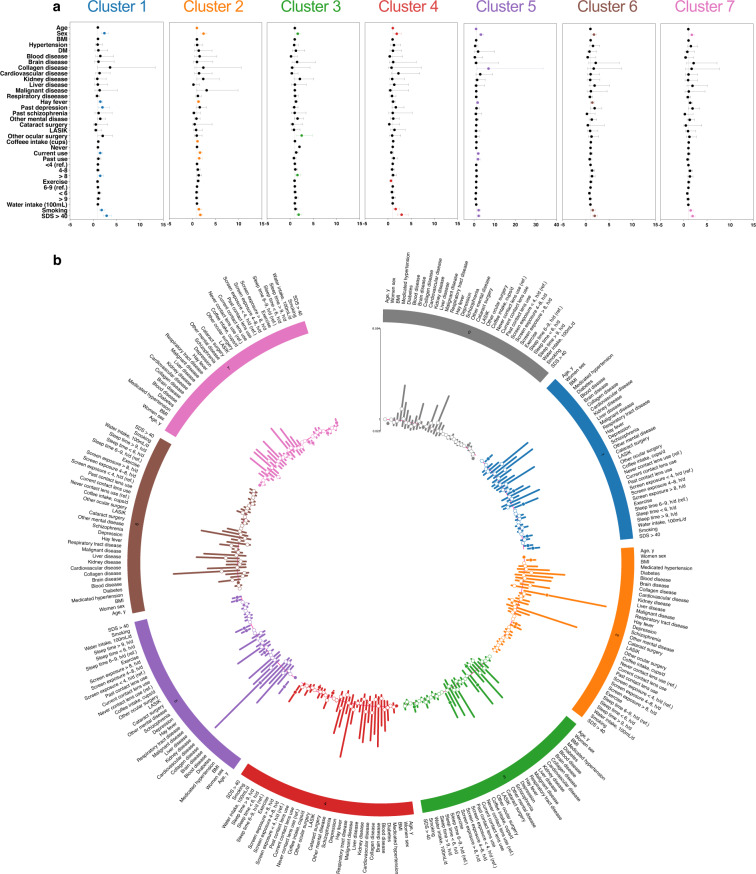
Risk stratification in each cluster. (**a**) Risk factors for each cluster in symptomatic DE compared with non-symptomatic DE. (**b**) Risk factors for each cluster in symptomatic DE compared with other clusters visualized in a circular layout. Abbreviation: DE dry eye.

Multivariable-adjusted logistic regression analyses were conducted to identify risk factors for each cluster among symptomatic versus non-symptomatic DE (J-OSDI total score <13), and among symptomatic DE versus data of all participants. Figure 6a presents the odds ratios (OR) of risk factors for each cluster in symptomatic DE compared with non-symptomatic DE (cluster 0) determined by fully adjusted logistic regression analysis (ORs and 95% confidence intervals, Supplementary Table 4). Younger age was identified as a risk factor in clusters 2, 5, and 6, compared with older age (clusters 4 and 7); collagen disease was identified as a risk factor only in cluster 5. Hay fever (clusters 1, 2, 5, and 6), current contact lens use (clusters 1, 2, and 5), screen exposure time >8 h (clusters 1 and 3), and smoking (clusters 1, 2, and 5) were identified as modifiable risk factors. Figure 6b depicts a Circos plot^30^, visualizing the ORs of each risk factor per cluster compared with others, determined by fully adjusted logistic regression analysis. Supplementary Table 5 shows the fully adjusted ORs of statistically significant factors for intercluster comparisons.

## Discussion

We demonstrated a novel multidimensional data-driven digital phenotyping methodology for DE using mHealth. This study successfully identified seven subgroups of symptomatic DE with distinct biological and behavioral profiles through multidimensional digital phenotyping for personalized DE intervention. Digital phenotyping through mHealth may elucidate the variability and heterogeneity of other diseases, actualizing P4 medicine in various healthcare fields.

DE is a highly variable disease with multifactorial influences and heterogeneous presentations^7,9^. However, a one-size-fits-all approach has been predominant, and minimal efforts have been made to personalize regimens according to individual factors^12^. Increasing reports and discussion regarding P4’s validity suggest preemptive and personalized healthcare both increase treatment and cost-effectiveness^26^, which necessitates a comprehensive dataset on patients’ subjective symptoms, lifestyle factors, and their environment. mHealth enables remote, longitudinal collection of various personalized data, including biosensor data, and enables participatory medical research through facilitating user comprehension, consent, and feedback^9,10^^,12^^,17,23,24,31^. The widespread prevalence of smartphones^32^ underscores mHealth’s potential to revolutionize the current facility-based healthcare by re-introducing it within free-living settings. Digital phenotyping and interventions gather vast comprehensive data on the user population and retain personalized aspects, allowing providers selective data application^33^. Additionally, mHealth facilitates disease monitoring through its PRO-integrated digital phenotyping as a remote diagnostic and interventional tool.

We investigated the effectiveness of smartphone applications as a digital phenotyping platform and were the first to explore MBI and blink frequency as digital phenotypes. Previous reports demonstrated a positive correlation between the MBI and the tear-film break-up time (TFBUT), and concomitant usage of the J-OSDI and MBI demonstrated potential as a non-invasive substitute^29,34^. In this study, symptomatic DE displayed a significant decrease in MBI, particularly in severe subgroups, suggesting MBI measurement could be highly specific in screening severe DE.

Effective DE-stratification strategies based on multi-dimensional symptom analyses and lifestyle factors were investigated. A dimension reduction of the aggregate DE subjective symptoms identified seven clusters of DE with distinct biological and behavioral profiles. Notably, clusters 2, 5, and 6 correlated with younger age, supporting the association between DE and aging^7^. However, our results may explain observed discrepancies in prior reports^7,17^. Traditional facility-based studies largely collected data in an equipped research facility—hospitals or otherwise—which led to a heavy reliance on participants to travel to a dedicated location. This inevitably excluded the younger, working-group population from participation, creating an inevitable selection bias^35^. However, a smartphone-based study enables a closer look at younger age groups^9,17,23,24,31^, which enables more robust hierarchical analyses to verify new disease subtypes. Unlike the traditional “cutoff-score” diagnoses^2,36^, this methodology stratifies groups of individual criteria, creating clusters of an originally singular disease to elucidate patterns of disease heterogeneity. For example, while cluster 1 displayed aggravation of all 12 symptoms, cluster 5 displayed vulnerability to environmental triggers (Fig. 3e and f). Recent studies showed an increasing prevalence of individuals with a short TFBUT-type DE, likely in part due to the aging and digital society^37^. The observed shortened MBI with higher J-OSDI total score in cluster 1, 2, and 5 show consistency with the previously reported short TFBUT-type DE, and the early identification of these subtypes may aid in guiding the appropriate tear-film oriented therapy^38^.

Modifiable risk factors could also be assessed and expeditiously addressed with interventions through smartphone applications. We demonstrated that a multidimensional data-driven analysis may aid in providing effective treatment by stratifying disease characteristics and tailoring the regimen according to the specific disease subgroup. For patients with similarities to cluster 1 with severe symptoms, individualized interventions to minimize pollen exposure, contact lens wear, on-screen time, smoking, and depression-related symptoms could be initiated (Fig. 6). With more studies reporting the connection between DE symptom aggravation and major depressive disorder, concomitantly monitoring mood-related symptoms may be helpful^24^. Previous studies support the value of digital phenotyping for identifying at-risk individuals, and accessible behavioral treatments through mHealth, either as a stand-alone or coached intervention^39,40^, may be valuable for the DE population^41^. mHealth dismantles the monolithic approach through early identification of a patient’s disease characteristics as the first step in patient-specific intervention.

mHealth yields high-volume, multidimensional integrative data, inherently difficult to analyze and interpret due to non-uniformity and noise. In part, the noise stems from the subjectivity and response bias that results from the reliance on self-reporting. Dimensional reduction greatly improves data interpretability by data-clustering on simple topological manifolds, with subsequent per-cluster analyses and visualization unveiling unique characteristics^42^. Here, we identified individual subjective symptoms through hierarchical heatmap analyses, visualized the per-cluster frequency of DE symptoms through chord diagrams, and analyzed each symptom’s correlation through co-occurrence network analysis, further increasing the stratified clusters’ interpretability.

Such pathology stratification strategies have been reserved for research on cancer and rare diseases’ treatment, largely focusing on fundamental hereditary disease etiologies^43,44^. We extended this strategy by applying stratification and individualization techniques in mHealth to actualize stratified medicine based on personal subjective symptoms and factors. Similar strategies could be applied for a myriad of diseases that require the interpretation of highly multidimensional integrative data through disease stratification. Additionally, the advantage of longitudinal data collection may be crucial when investigating mechanisms of disease pathogenesis and progression.

True disease states are not determined by thresholds of observed biomarkers but constitute a spectrum between healthy and severely affected states. Therefore, the ability to specify one’s disease locus is essential in determining proper intervention. Here, UMAP, hierarchical clustering, chord diagrams, co-occurrence network analyses, and Circos plot successfully stratified and visualized the interactions of 12 key DE symptoms and risk factors. Furthermore, immediate feedbacks, including dryness evaluations via the J-OSDI, blink measurements, and lifestyle assessments, were provided, both as an interventional tool and an incentive to motivate user participation (Fig. 1a, b). This may help facilitate the implementation and public uptake of novel technologies^33,45^. The role of mHealth technology has been gaining global attention, evidenced by the “Be He@lthy, Be Mobile (BHBM)” initiative of the World Health Organization and position papers of renowned journals, including the American College of Allergy and Asthma and Immunology (ACAAI) and the European Academy of Allergy and Clinical Immunology (EAACI)^46–48^. As a notable example, a recent study demonstrated the effectiveness of an mHealth-driven stratification technique on the heterogeneous symptoms of hay fever in creating a foundation for individualized therapeutic interventions^18^. mHealth is positioned to seamlessly fit within the lifestyle of the global population, making it ideal in actualizing healthcare within one’s life and providing early identification and intervention of diseases.

Our study has several limitations. First, this cohort contains age-related selection bias, socioeconomic factors, and educational level, and its initial release was solely on the iOS platform^9^. Aging is associated with lower rates of smartphone use, partially due to cognitive changes and reduced fine motor control^49^. However, the major cause of this barrier was a lack of training, underscoring the social efforts needed to introduce modern devices to older populations. In addition, design guidelines should address older adults’ diminishing cognition skills, physical ability, and motivational barriers^50^. Regarding our study, an Android version was released on September 3, 2020, and socioeconomic and educational surveys were added to track their influences on the results. Nonetheless, to minimize any potential bias, both iOS and Android-supported apps should be used from inception for future studies. Additionally, health-seeking behavior may have limited this study’s generalizability, as participants who actively participated in the study were more likely to have DE-related symptoms. Additionally, the demographics of our participants suggest that the study results are more applicable to the younger population, likely due to the nature of an mHealth study. Therefore, careful external validation may be needed to correlate this study’s findings to previous studies that are based on the older population^7^. Second, self-administered questionnaires may introduce self-reporting bias. However, the J-OSDI questionnaire was validated through paper-based and DryEyeRhythm-based questionnaires; therefore, the results of self-administered questionnaires were deemed valid^9^. Third, this study identified symptomatic DE solely based on the J-OSDI questionnaire and without clinical examinations. A previous study reports that a considerable discrepancy may exist between subjectively reported symptoms and objectively measured clinical findings of DE^51^. Therefore, this study may be susceptible to false-positive findings, and the authors ask the readers to be mindful of the magnitude of self-reported data in this study when interpreting the results. However, recent reports suggest that MBI measurement is an effective non-invasive alternative to tear-film break-up time, and smartphone camera-based blink biosensors may be a valid DE diagnostic tool^29,34^. Finally, regarding sample size, this study was based on the best available sample size collected to date. Some stratified groups were small in size and, therefore, some variables might not have been identified as statistically significant.

While addressing the existing biases, future studies should assess the scalability, applicability, and practicality of mHealth by further extracting key digital phenotypes and demonstrating their value for other diseases. While considerable efforts have been made in numerous fields, including psychiatry^33^, timely integration of mHealth may be crucial for equity in healthcare regardless of specialty, especially during a pandemic. Support for diverse operating systems, simplification of the user interface, population training, and medical data security are all topics that require thorough discussion. With increasing evidence, including our own, of the value offered by mHealth instituting non-traditional methodologies, emphasis on broad usage and effective implementation may be paramount for a prompt uptake of mHealth and actualization of P4 medicine.

In conclusion, we developed a smartphone-based digital phenotyping strategy for heterogeneous DE presentations. This sequence of data collection and analysis that allows for stratification and visualization of diseases might be valuable in unveiling their heterogeneity and fundamental pathology and increasing the quality of healthcare through establishing a robust foundation for P4 medicine.

## Methods

### DryEyeRhythm smartphone application

The DryEyeRhythm app^9^ was initially developed using Apple Inc.’s (Cupertino, CA, USA) open-source framework, ResearchKit^52^. The DryEyeRhythm app was released in February 2016 for iOS and September 2020 for Android under a consignment contract with Juntendo University Graduate School of Medicine, Tokyo, Japan, and InnoJin, Inc., Tokyo, Japan. It is freely available on Apple’s App Store and Google Play.

### Study design and participants

This cross-sectional crowdsourced study was conducted using the iOS version of DryEyeRhythm (Fig. 1a). This study was conducted between November 2, 2016, and September 30, 2019. Previous studies have reported the enrollment process^9,17,23,24^. Key performance indicators, including impressions, downloads, and sessions, were measured using App Analytics within the App Store Connect. Electronic informed consent for participation was obtained from all users. We included DryEyeRhythm users in Japan, excluding duplicate and incomplete user data. This study was approved by the Independent Ethics Committee of Juntendo University Faculty of Medicine (approval numbers: 16-078 and 16-152) per the Declaration of Helsinki. The methodology and results of this survey are reported as per the CHERRIES reporting guidelines^53^.

### User-data collection

Figure 1b depicts the DryEyeRhythm’s user experience. This method initially collects electronic informed consent for participation after explaining the study’s nature and possible consequences. The data collection parameters included questions about demographics, medical history, lifestyle questionnaire, blink sensing, and daily subjective symptoms (Supplementary Table 6)^9,17,23,24^. Participants also reported daily subjective symptoms (Supplementary Table 5) and answered, among others, the J-OSDI and the Zung SDS questionnaires^14,15^ for DE and depression^54^, respectively (Supplementary Tables 7 and 8).

### Blink sensing using a blink-detection application programming interface

Blinking was measured with the users’ own smartphone cameras and CIFaceFeature in the iOS interface for facial detection (available at https://developer.apple.com/documentation/coreimage/cifacefeature). Blinking was defined as opening both eyes followed by their closure. MBI was defined as the maximum inter-blink duration per 30-s trial^29,34^.

### Symptomatic DE ascertainment

DE’s subjective symptoms were assessed using the 12-item J-OSDI questionnaire (Supplementary Table 7) with three subscales: ocular symptoms, visual functioning, and environmental triggers^14^. Each response was recorded on a five-point scale [“None of the time” (0) / “All of the time” (4)], with “N/A” for questions not applicable to the user. The total score was reported on a 100-point scale, determining DE symptom severity (0–12: normal; 13–22: mild; 23–33: moderate; and 33–100: severe). The J-OSDI is validated in Japan as the Japanese version of the OSDI^15^; the DryEyeRhythm-based J-OSDI is validated based on the paper-based J-OSDI^9^.

We classified the study participants into two groups^17^: non-symptomatic DE (J-OSDI total score <13) and symptomatic DE (J-OSDI total score ≥13).

### Zung SDS

The internationally used 20-item SDS evaluates depression symptoms (Supplementary Table 8)^54^ and is validated in Japan^55,56^. Each item is rated on the following 4-point Likert scale: “A little of the time,” “Some of the time,” “Good part of the time,” and “Most of the time.” Total scores range from 20 to 80; a score ≥40 suggests depression^57^.

### Symptom-based stratification

Among symptomatic individuals with DE, normalized maximum eigengap values estimated the number of clusters during spectral clustering^27^. The UMAP with spectral clustering for dimension reduction was used to depict the stratification of subjective symptoms of symptomatic DE based on the 12 J-OSDI questions. UMAP was performed by the umap-learn python package (version 0.4.6)^28^.

### Hierarchical clustering heatmap

A hierarchical clustering heatmap was constructed using the matplotlib module (version 0.9.0, Python 3). Stratified clusters identified by the UMAP are shown on the left side of the heatmap as a color bar. The dendrograms’ clustering for each of the J-OSDI’s 12 items is shown on the top of the heatmap. Heatmaps are colored by the score of the J-OSDI items (0–4) with a color bar legend at the left top.

### Chord diagram

A chord diagram illustrates inter-relationships between each J-OSDI item in each cluster. Each of the 12 items was assigned to one of eight groups for illustration. Colors are based on the participants’ clustered group. Lines represent the connections between the participants’ clustered group (at the base of the lines in the lower half of the circle) and the J-OSDI items (at the base of the lines in the upper half of the circle). Line thickness represents the participants’ number. The Chord diagrams are colored by the threshold score of the J-OSDI items (0–4) with a color bar legend at the right. Plots were created in R version 4.0.3 (2020-10-10) using the circlize package (version 0.4.11)^58^.

### Co-occurrence network analysis

Co-occurrence network analysis found cluster-based significant correlations between each J-OSDI item. Co-occurrence network analyses were conducted with the wTO metric, normalizing all shared correlations between a pair of parameters. The wTO metrics were calculated using R version’s 4.0.3 wTO package (2020-10-10). All item networks were constructed using Pearson correlation using 250 bootstraps^59^. Networks were filtered for a Benjamini and Hochberg adjusted *P* < 0.001. Significant links were visualized using the NetVis function in the wTO package.

In the co-occurrence network analysis representations, nodes (12 items of the J-OSDI) are represented as circles; node links (edges) are represented as lines. Each node size is proportional to the frequency of each J-OSDI-defined DE symptom. Edges show pairwise comparison strength for each J-OSDI item domain interrelation. A thicker edge across all networks implies a stronger association, facilitating strength comparison. Purple and green edges represent positive and negative interconnections, respectively.

### Multivariate logistic regression analysis

Multivariable-adjusted logistic regression analyses identified risk factors for each cluster among symptomatic versus non-symptomatic DE (J-OSDI total score <13), and among symptomatic DE versus data of all participants. The covariates were selected based on previously established methodology^7,9,23,24^. The Circos plot of risk factors for each cluster among the symptomatic DE versus the data of all participants was visualized using the EpiCircos (available at https://github.com/mattlee821/EpiViz)^30^.

### Statistical analysis

Participant characteristics were compared between symptomatic and asymptomatic DE and each of their cluster. Continuous variables are presented as median (interquartile range [IQR]) for non-normally distributed factors, based on Shapiro-Wilk tests; categorical variables are presented as a percentage. We conducted χ^2^ and Mann-Whitney U tests for categorical and non-normally distributed continuous variables.

All data were analyzed with STATA version 15 (Stata Corp, College Station, TX, USA). Moreover, a heatmap for the geographic distribution of the participants in Japan (Fig. 2e) was constructed using the matplotlib module (version 0.9.0, Python 3).

### Reporting summary

Further information on research design is available in the Nature Research Reporting Summary linked to this article.

## Supplementary information

Supplementary Information

Reporting Summary

## Data Availability

All data are available in the main text or the supplementary materials.

## Code availability

The code that supports the findings of this study are available from the corresponding author upon reasonable request. Analysis to process and analyze data was generated with Python 3, R version’s 4.0.3, and STATA version 15.
