# Supplementary material for: Smartphone-based digital phenotyping for dry eye toward P4 medicine: a crowdsourced cross-sectional study
Source: NPJ Digit Med. 2021 Dec 20;4:171. doi: 10.1038/s41746-021-00540-2 (PMC8688467; doi:10.1038/s41746-021-00540-2)
Supplement: Supplementary file 1 — Supplementary Information [file 41746_2021_540_MOESM1_ESM.pdf]

SUPPLEMENTARY FILES

Supplementary Table 1. Sensitivity analysis between included and excluded participants.

	Included		Excluded		
	n=3,593 (45.8%)	Response number	n=4,144 (54.2%)	Response number	*P*-value
Demographic characteristics					
Age (years), median [IQR]	27 [20–41]	3,593	27 [20–39]	4,141	0.078
Age category (years), number (%)		3,593		4,141	0.043
<20	896 (24.9)		1,012 (24.4)		
20–30	1,102 (30.7)		1,368 (33.0)		
30–40	612 (17.0)		749 (18.1)		
40–50	564 (15.7)		575 (13.9)		
50–60	312 (8.7)		332 (8.0)		

≥60	107 (3.0)		105 (2.5)		
Women, number (%)	2,147 (59.8)	3,593	2,142 (51.7)	4,141	< 0.001
Height (cm), median [IQR]	162 [157–170]	3,593	164 [158–171]	4,141	< 0.001
Body weight (kg), median [IQR]	57 [50–66}	3,593	58 [50–67]	4,141	0.020
Body mass index[a], median [IQR]	21.4 [19.5–24.0]	3,593	21.4 [19.5–23.8]	4,141	0.400
Medical history					
Medicated					
Hypertension, number (%)	152 (4.2)	3,593	124 (3.0)	4,141	0.003
Diabetes, number (%)	66 (1.8)	3,593	62 (1.5)	4,141	0.243
Systemic diseases, number (%)					

Blood disease	36 (1.0)	3,593	33 (0.8)	4,141	0.339
Brain disease	30 (0.8)	3,593	24 (0.6)	4,141	0.179
Collagen disease	32 (0.9)	3,593	21 (0.5)	4,141	0.041
Heart disease	70 (2.0)	3,593	76 (1.8)	4,141	0.716
Kidney disease	65 (1.8)	3,593	42 (1.0)	4,141	0.003
Liver disease	50 (1.4)	3,593	51 (1.2)	4,141	0.536
Malignant tumor	27 (0.8)	3,593	36 (0.9)	4,141	0.565
Respiratory disease	257 (7.2)	3,593	228 (5.5)	4,141	0.003
Hay fever, number (%)	1,817 (50.6)	3,593	2,080 (50.2)	4,141	0.765
Mental illness, number (%)					
Depression	167 (4.7)	3,593	137 (3.3)	4,141	0.002
Schizophrenia	31 (0.9)	3,593	29 (0.7)	4,141	0.417
Others	191 (5.3)	3,593	149 (3.6)	4,141	< 0.001

Past diagnosis of dry eye disease, number (%)	894 (24.9)	3,593	975 (23.6)	4,141	0.171
Ophthalmic surgery, number (%)					
Cataract surgery	21 (0.6)	3,593	18 (0.4)	4,141	0.354
LASIK	63 (1.8)	3,593	99 (2.4)	4,141	0.051
Others	105 (2.9)	3,593	118 (2.9)	4,141	0.849
Eye drop use, number (%)	890 (24.8)	3,593	959 (23.2)	4,141	0.097
Lifestyle habits					
Coffee (cups per day), median [IQR]	0 [0–2]	3,593	0 [0–2]	884	0.009
Contact lens use, number (%)		3,593		4,141	0.861
Negative	1,753 (48.8)		1,997 (48.2)		

Current use	1,400 (39.0)		1,638 (39.6)		
Past use	440 (12.3)		506 (12.2)		
Screen exposure time (hours per day), median [IQR]	6 [4–10]	3,593	6 [4–10]	884	0.869
Screen exposure category (hours per day), number (%)		3,593		884	0.607
<4	637 (17.7)		169 (19.1)		
4–8	1,890 (52.6)		461 (52.5)		
>8	1,066 (29.7)		254 (28.7)		
Periodic exercise, number (%)	2,292 (63.8)	3,593	2,748 (66.4)	4,141	0.018

Periodic exercise (hours per week), median [IQR]	1 [0–3]	3,593	1 [0–3]	4,141	0.005
Sleeping time (hours per day), median [IQR]	7 [6–8.5]	3,593	7 [5.7–8.5]	884	0.134
Sleeping time category (hours per day), number (%)		3,593		884	0.014
<6	1,031 (28.7)		294 (33.3)		
6–9	1,924 (53.6)		429 (48.5)		
>9	638 (17.8)		161 (18.2)		
Smoking, number (%)	1,005 (28.0)	3,593	1,143 (27.6)	4,141	0.718
Water intake (100 mL per day), median [IQR]	8 [4–10]	3,593	8 [4–10]	884	0.212

Subjective symptoms					
Daily subjective symptoms					
Eye itching, (0, not at all to 10, very itchy), median [IQR]	2 [0–4]	3,593	2 [0–5]	884	0.222
Asthenopia, number (%)	2,168 (60.3)	3,5933,593	471 (53.3)	884	< 0.001
Headache, (0, not at all to 10, very painful), median [IQR]	1 [0–3]	3,593	1 [0–3]	884	0.753
Mental fatigue, number (%)	995 (27.7)	3,593	198 (22.4)	884	0.001
Stiffness and pain of body axis muscles, number (%)	1,880 (52.3)	3,593	375 (42.4)	884	< 0.001

Stress, (0, not at all to 10, I feel very stressed), median [IQR]	5 [2–7]	3,593	4 [2–7]	884	0.239
OSDI, (0–100), median [IQR]					
OSDI total score	22.9 [12.5–36.1]	3,593	20.8 [11.4–33.3]	4,997	0.006
Ocular symptoms	25 [15–40]	3,593	25 [15–35]	4,144	< 0.001
Vision-related function	12.5 [0–25]	3,567	8.3 [0–25]	3,891	0.018
Environmental triggers	25 [8.3–50]	3,574	25 [8.3–41.7]	3,887	< 0.001
SDS					

SDS total score, (20–80), median [IQR]	44 [37–51]	3,593	44 [37–52]	1,631	0.154
Depressive symptoms score ≥40, number (%)	2,362 (65.7)	3,593	1,109 (68.0)	1,631	0.110

IQR: interquartile range; LASIK: laser-assisted *in situ* keratomileusis; OSDI: Japanese version of the ocular surface disease index; SDS: Zung Self-rating Depression Scale

[a] Calculated as weight in kilograms divided by height in meters squared.

Supplementary Table 2. Participants' characteristics.

Demographic characteristics	Non-symptomatic DE n=974 (27.1)	Symptomatic DE n=2,619 (72.9)	*P*-value	Overall n=3,593 (100)
Age (years), median [IQR]	30 [20–44]	26 [19–40]	< 0.001	27 [20–41]
Age category (years), number (%)			< 0.001	
<20	214 (22.0)	682 (26.0)		896 (24.9)
20–30	263 (27.0)	839 (32.0)		1,102 (30.7)
30–40	190 (19.5)	422 (16.1)		612 (17.0)
40–50	174 (17.9)	390 (14.9)		564 (15.7)
50–60	98 (10.1)	214 (8.2)		312 (8.7)
≥60	35 (3.6)	72 (2.8)		107 (3.0)
Women, number (%)	446 (45.8)	1,701 (65.0)	< 0.001	2,147 (59.8)

Height (cm), median [IQR]	165 [158–172]	161 [156–168]	< 0.001	162 [157–170]
Body weight (kg), median [IQR]	60 (52–68)	56 (50–65)	< 0.001	57 [50–66]
Body mass index[a], median [IQR]	21.8 (19.8–24.2)	21.3 (19.4–23.9)	0.001	21.4 [19.5–24.0]
Medical history				
Medicated hypertension, number (%)	46 (4.7)	106 (4.1)	0.371	152 (4.2)
Diabetes, number (%)	22 (2.3)	44 (1.7)	0.251	66 (1.8)
Systemic diseases, number (%)				
Blood disease	12 (1.2)	24 (0.9)	0.398	36 (1.0)
Brain disease	9 (0.9)	21 (0.8)	0.720	30 (0.8)
Collagen disease	5 (0.5)	27 (1.0)	0.142	32 (0.9)
Heart disease	16 (1.6)	54 (2.1)	0.419	70 (2.0)
Kidney disease	13 (1.3)	52 (2.0)	0.193	65 (1.8)
Liver disease	13 (1.3)	37 (1.4)	0.859	50 (1.4)
Malignant tumor	8 (0.8)	19 (0.7)	0.767	27 (0.8)

Respiratory disease	58 (6.0)	199 (7.6)	0.089	257 (7.2)
Hay fever, number (%)	447 (45.9)	1,370 (52.3)	0.001	1,817 (50.6)
Mental illness, number (%)				
Depression	29 (3.0)	138 (5.3)	0.004	167 (4.7)
Schizophrenia	9 (0.9)	22 (0.8)	0.809	31 (0.9)
Others	32 (3.3)	159 (6.1)	0.001	191 (5.3)
Past diagnosis of dry eye disease, number (%)	141 (14.5)	753 (28.8)	< 0.001	894 (24.9)
Ophthalmic surgery, number (%)				
Cataract surgery	10 (1.0)	11 (0.4)	0.034	21 (0.6)
LASIK	23 (2.4)	40 (1.5)	0.090	63 (1.8)
Others	24 (2.5)	81 (3.1)	0.320	105 (2.9)
Eye drop use, number (%)	147 (15.1)	743 (28.4)	< 0.001	890 (24.8)
Lifestyle habits				
Coffee (cups per day), median [IQR]	1 [0–2]	0 [0–2]	0.402	0 [0–2]

Contact lens use, number (%)			< 0.001	
Negative	537 (55.1)	1,216 (46.4)		1,753 (48.8)
Current use	307 (31.5)	1,093 (41.7)		1,400 (39.0)
Past use	130 (13.4)	310 (11.8)		440 (12.3)
Screen exposure time (hours per day), median [IQR]	6 [4–10]	6 [4–10]	< 0.001	6 [4–10]
Periodic exercise, number (%)	645 (66.2)	1,647 (62.9)	0.064	2,292 (63.8)
Periodic exercise (hours per week), median [IQR]	1 [0–4]	1[(0–3)	0.020	1 [0–3]
Sleeping time (hours per day), median [IQR]	7 [6–8.5]	7 [6–8.5]	0.490	7 [6–8.5]
Smoking, number (%)	245 (25.2)	760 (29.0)	0.022	1,005 (28.0)
Water intake (100 mL per day), median [IQR]	8 [4–10]	8 [4–10]	0.130	8 [4–10]
Subjective symptoms				
Daily subjective symptoms				

Eye itching, (0, not at all to 10, very itchy), median [IQR]	0 [0–2]	2 [0–5]	< 0.001	2 [0–4]
Asthenopia, number (%)	395 (40.6)	1,773 (67.7)	< 0.001	2,168 (60.3)
Headache, (0, not at all to 10, very painful), median [IQR]	0 [0–2]	1 [0–4]	< 0.001	1 [0–3]
Mental fatigue, number (%)	172 (17.7)	823 (31.4)	< 0.001	995 (27.7)
Stiffness and pain of body axis muscles, number (%)	387 (39.7)	1,493 (57.0)	< 0.001	1,880 (52.3)
Stress, (0, not at all to 10, I feel very stressed), median [IQR]	3 [2–6]	5 [3–7]	< 0.001	5 [2–7]
OSDI, (0–100), median [IQR]				
OSDI total score	8.3 [4.5–10.4]	29.2 [20.8–41.7]	< 0.001	22.9 [12.5–36.1]
Ocular symptoms	10 [5–15]	35 [25–45]	< 0.001	25 [15–40]
Vision-related function	0 [0–6.3]	18.8 [6.3–31.3]	< 0.001	12.5 [0–25]

Environmental triggers	0 [0–16.7]	33.3 [16.7–58.3]	< 0.001	25 [8.3–50]
SDS				
SDS total score, (20–80), median [IQR]	39.5 [34–46]	45 [38–52]	< 0.001	44 [37–51]
Depressive symptoms score ≥40, number (%)	487 (50.0)	1,875 (71.6)	< 0.001	2,362 (65.7)

DE: dry eye; IQR: interquartile range; LASIK: laser-assisted *in situ* keratomileusis; OSDI: ocular surface disease index; SDS: Zung Self-rating Depression Scale.

[a] Calculated as weight in kilograms divided by height in meters squared.

Supplementary Table 3. wTO and Pearson coefficient values for network analysis.

a. Cluster 0

Node 1	Node 2	wTO with positive and negative signs	*P*-value	Adjusted *P*-value	Pearson coefficient value
J-OSDI 1	J-OSDI 2	0.040	< 0.001	< 0.001	0.052
J-OSDI 1	J-OSDI 3	0.001	< 0.001	< 0.001	-0.017
J-OSDI 1	J-OSDI 4	0.028	< 0.001	< 0.001	0.038
J-OSDI 1	J-OSDI 5	-0.005	< 0.001	< 0.001	-0.015
J-OSDI 1	J-OSDI 6	-0.055	< 0.001	< 0.001	-0.077
J-OSDI 1	J-OSDI 7	-0.020	< 0.001	< 0.001	-0.014
J-OSDI 1	J-OSDI 8	-0.032	< 0.001	< 0.001	-0.036
J-OSDI 1	J-OSDI 9	-0.022	< 0.001	< 0.001	-0.027
J-OSDI 1	J-OSDI 0	-0.019	< 0.001	< 0.001	0.007
J-OSDI 1	J-OSDI 11	-0.068	< 0.001	< 0.001	-0.069
J-OSDI 1	J-OSDI 12	-0.072	< 0.001	< 0.001	-0.075
J-OSDI 2	J-OSDI 3	0.151	< 0.001	< 0.001	0.184
J-OSDI 2	J-OSDI 4	-0.035	< 0.001	< 0.001	-0.012
J-OSDI 2	J-OSDI 5	-0.062	< 0.001	< 0.001	-0.058
J-OSDI 2	J-OSDI 6	-0.092	< 0.001	< 0.001	-0.107
J-OSDI 2	J-OSDI 7	-0.102	< 0.001	< 0.001	-0.117
J-OSDI 2	J-OSDI 8	-0.083	< 0.001	< 0.001	-0.076

J-OSDI 2	J-OSDI 9	-0.065	< 0.001	< 0.001	-0.064
J-OSDI 2	J-OSDI 10	0.019	< 0.001	< 0.001	-0.026
J-OSDI 2	J-OSDI 11	0.060	< 0.001	< 0.001	0.072
J-OSDI 2	J-OSDI 12	0.047	< 0.001	< 0.001	0.047
J-OSDI 3	J-OSDI 4	-0.007	< 0.001	< 0.001	0.019
J-OSDI 3	J-OSDI 5	-0.039	< 0.001	< 0.001	-0.036
J-OSDI 3	J-OSDI 6	-0.059	< 0.001	< 0.001	-0.051
J-OSDI 3	J-OSDI 7	-0.050	< 0.001	< 0.001	-0.041
J-OSDI 3	J-OSDI 8	-0.059	< 0.001	< 0.001	-0.060
J-OSDI 3	J-OSDI 9	-0.027	< 0.001	< 0.001	-0.019
J-OSDI 3	J-OSDI 10	0.041	< 0.001	< 0.001	0.056
J-OSDI 3	J-OSDI 11	0.032	< 0.001	< 0.001	0.015
J-OSDI 3	J-OSDI 12	0.014	< 0.001	< 0.001	-0.013
J-OSDI 4	J-OSDI 5	0.225	< 0.001	< 0.001	0.309
J-OSDI 4	J-OSDI 6	0.129	< 0.001	< 0.001	0.151
J-OSDI 4	J-OSDI 7	0.038	< 0.001	< 0.001	0.041
J-OSDI 4	J-OSDI 8	0.030	< 0.001	< 0.001	0.013
J-OSDI 4	J-OSDI 9	0.060	< 0.001	< 0.001	0.034
J-OSDI 4	J-OSDI 10	-0.108	< 0.001	< 0.001	-0.090
J-OSDI 4	J-OSDI 11	-0.153	< 0.001	< 0.001	-0.145
J-OSDI 4	J-OSDI 12	-0.146	< 0.001	< 0.001	-0.139
J-OSDI 5	J-OSDI 6	0.141	< 0.001	< 0.001	0.172
J-OSDI 5	J-OSDI 7	0.019	< 0.001	< 0.001	-0.022

J-OSDI 5	J-OSDI 8	0.051	< 0.001	< 0.001	0.044
J-OSDI 5	J-OSDI 9	0.115	< 0.001	< 0.001	0.123
J-OSDI 5	J-OSDI 10	-0.092	< 0.001	< 0.001	-0.068
J-OSDI 5	J-OSDI 11	-0.125	< 0.001	< 0.001	-0.114
J-OSDI 5	J-OSDI 12	-0.124	< 0.001	< 0.001	-0.117
J-OSDI 6	J-OSDI 7	0.091	< 0.001	< 0.001	0.102
J-OSDI 6	J-OSDI 8	0.072	< 0.001	< 0.001	0.065
J-OSDI 6	J-OSDI 9	0.050	< 0.001	< 0.001	0.025
J-OSDI 6	J-OSDI 10	-0.081	< 0.001	< 0.001	-0.084
J-OSDI 6	J-OSDI 11	-0.089	< 0.001	< 0.001	-0.065
J-OSDI 6	J-OSDI 12	-0.081	< 0.001	< 0.001	-0.068
J-OSDI 7	J-OSDI 8	0.078	< 0.001	< 0.001	0.087
J-OSDI 7	J-OSDI 9	0.029	< 0.001	< 0.001	0.028
J-OSDI 7	J-OSDI 10	-0.035	< 0.001	< 0.001	-0.036
J-OSDI 7	J-OSDI 11	-0.040	< 0.001	< 0.001	-0.034
J-OSDI 7	J-OSDI 12	-0.016	< 0.001	< 0.001	0.014
J-OSDI 8	J-OSDI 9	0.041	< 0.001	< 0.001	0.039
J-OSDI 8	J-OSDI 10	-0.012	< 0.001	< 0.001	0.005
J-OSDI 8	J-OSDI 11	-0.029	< 0.001	< 0.001	-0.020
J-OSDI 8	J-OSDI 12	-0.021	< 0.001	< 0.001	-0.011
J-OSDI 9	J-OSDI 10	-0.011	< 0.001	< 0.001	0.009
J-OSDI 9	J-OSDI 11	-0.033	< 0.001	< 0.001	-0.006
J-OSDI 9	J-OSDI 12	-0.047	< 0.001	< 0.001	-0.042

J-OSDI 10	J-OSDI 11	0.264	< 0.001	< 0.001	0.304
J-OSDI 10	J-OSDI 12	0.214	< 0.001	< 0.001	0.208
J-OSDI 11	J-OSDI 12	0.311	< 0.001	< 0.001	0.430

b. Cluster 1

Node 1	Node 2	wTO with positive and negative signs	*P*-value	Adjusted *P*-value	Pearson coefficient value
J-OSDI1	J-OSDI 2	0.152	< 0.001	< 0.001	0.148
J-OSDI 1	J-OSDI 3	0.166	< 0.001	< 0.001	0.198
J-OSDI 1	J-OSDI 4	0.134	< 0.001	< 0.001	0.101
J-OSDI 1	J-OSDI 5	0.176	< 0.001	< 0.001	0.151
J-OSDI 1	J-OSDI 6	0.184	< 0.001	< 0.001	0.193
J-OSDI 1	J-OSDI 7	-0.011	< 0.001	< 0.001	-0.026
J-OSDI 1	J-OSDI 8	0.125	< 0.001	< 0.001	0.104
J-OSDI 1	J-OSDI 9	0.117	< 0.001	< 0.001	0.084
J-OSDI 1	J-OSDI 0	0.195	< 0.001	< 0.001	0.251
J-OSDI 1	J-OSDI 11	0.156	< 0.001	< 0.001	0.155
J-OSDI 1	J-OSDI 12	0.119	< 0.001	< 0.001	0.068
J-OSDI 2	J-OSDI 3	0.263	< 0.001	< 0.001	0.356
J-OSDI 2	J-OSDI 4	0.151	< 0.001	< 0.001	0.145
J-OSDI 2	J-OSDI 5	0.144	< 0.001	< 0.001	0.097

J-OSDI 2	J-OSDI 6	0.080	< 0.001	< 0.001	-0.002
J-OSDI 2	J-OSDI 7	-0.056	< 0.001	< 0.001	-0.067
J-OSDI 2	J-OSDI 8	0.061	< 0.001	< 0.001	0.021
J-OSDI 2	J-OSDI 9	0.060	< 0.001	< 0.001	0.014
J-OSDI 2	J-OSDI 10	0.120	< 0.001	< 0.001	0.104
J-OSDI 2	J-OSDI 11	0.132	< 0.001	< 0.001	0.136
J-OSDI 2	J-OSDI 12	0.082	< 0.001	< 0.001	0.044
J-OSDI 3	J-OSDI 4	0.165	< 0.001	< 0.001	0.190
J-OSDI 3	J-OSDI 5	0.165	< 0.001	< 0.001	0.141
J-OSDI 3	J-OSDI 6	0.119	< 0.001	< 0.001	0.079
J-OSDI 3	J-OSDI 7	-0.087	< 0.001	< 0.001	-0.123
J-OSDI 3	J-OSDI 8	0.119	< 0.001	< 0.001	0.125
J-OSDI 3	J-OSDI 9	0.107	< 0.001	< 0.001	0.081
J-OSDI 3	J-OSDI 10	0.118	< 0.001	< 0.001	0.105
J-OSDI 3	J-OSDI 11	0.120	< 0.001	< 0.001	0.113
J-OSDI 3	J-OSDI 12	0.106	< 0.001	< 0.001	0.082
J-OSDI 4	J-OSDI 5	0.304	< 0.001	< 0.001	0.458
J-OSDI 4	J-OSDI 6	0.188	< 0.001	< 0.001	0.168
J-OSDI 4	J-OSDI 7	-0.078	< 0.001	< 0.001	-0.071
J-OSDI 4	J-OSDI 8	0.128	< 0.001	< 0.001	0.107
J-OSDI 4	J-OSDI 9	0.147	< 0.001	< 0.001	0.119
J-OSDI 4	J-OSDI 10	0.119	< 0.001	< 0.001	0.073
J-OSDI 4	J-OSDI 11	0.111	< 0.001	< 0.001	0.073

J-OSDI 4	J-OSDI 12	0.104	< 0.001	< 0.001	0.068
J-OSDI 5	J-OSDI 6	0.231	< 0.001	< 0.001	0.338
J-OSDI 5	J-OSDI 7	-0.072	< 0.001	< 0.001	-0.124
J-OSDI 5	J-OSDI 8	0.159	< 0.001	< 0.001	0.066
J-OSDI 5	J-OSDI 9	0.198	< 0.001	< 0.001	0.207
J-OSDI 5	J-OSDI 10	0.163	< 0.001	< 0.001	0.184
J-OSDI 5	J-OSDI 11	0.154	< 0.001	< 0.001	0.157
J-OSDI 5	J-OSDI 12	0.151	< 0.001	< 0.001	0.096
J-OSDI 6	J-OSDI 7	0.016	< 0.001	< 0.001	0.076
J-OSDI 6	J-OSDI 8	0.235	< 0.001	< 0.001	0.265
J-OSDI 6	J-OSDI 9	0.237	< 0.001	< 0.001	0.275
J-OSDI 6	J-OSDI 10	0.197	< 0.001	< 0.001	0.228
J-OSDI 6	J-OSDI 11	0.174	< 0.001	< 0.001	0.176
J-OSDI 6	J-OSDI 12	0.171	< 0.001	< 0.001	0.152
J-OSDI 7	J-OSDI 8	-0.032	< 0.001	< 0.001	-0.034
J-OSDI 7	J-OSDI 9	-0.062	< 0.001	< 0.001	-0.099
J-OSDI 7	J-OSDI 10	0.092	< 0.001	< 0.001	0.184
J-OSDI 7	J-OSDI 11	0.065	< 0.001	< 0.001	0.120
J-OSDI 7	J-OSDI 12	0.011	< 0.001	< 0.001	-0.024
J-OSDI 8	J-OSDI 9	0.270	< 0.001	< 0.001	0.377
J-OSDI 8	J-OSDI 10	0.126	< 0.001	< 0.001	0.099
J-OSDI 8	J-OSDI 11	0.092	< 0.001	< 0.001	0.056
J-OSDI 8	J-OSDI 12	0.079	< 0.001	< 0.001	0.027

J-OSDI 9	J-OSDI 10	0.123	< 0.001	< 0.001	0.107
J-OSDI 9	J-OSDI 11	0.092	< 0.001	< 0.001	0.041
J-OSDI 9	J-OSDI 12	0.102	< 0.001	< 0.001	0.084
J-OSDI 10	J-OSDI 11	0.214	< 0.001	< 0.001	0.281
J-OSDI 10	J-OSDI 12	0.181	< 0.001	< 0.001	0.166
J-OSDI 11	J-OSDI 12	0.267	< 0.001	< 0.001	0.363

c. Cluster 2

Node 1	Node 2	wTO with positive and negative signs	*P*-value	Adjusted *P*-value	Pearson coefficient value
J-OSDI 1	J-OSDI 2	-0.008	< 0.001	< 0.001	-0.040
J-OSDI 1	J-OSDI 3	0.039	< 0.001	< 0.001	0.056
J-OSDI 1	J-OSDI 4	0.019	< 0.001	< 0.001	-0.001
J-OSDI 1	J-OSDI 5	0.072	< 0.001	< 0.001	0.099
J-OSDI 1	J-OSDI 6	-0.022	< 0.001	< 0.001	-0.057
J-OSDI 1	J-OSDI 7	0.044	< 0.001	< 0.001	0.056
J-OSDI 1	J-OSDI 8	-0.029	< 0.001	< 0.001	-0.058
J-OSDI 1	J-OSDI 9	0.055	< 0.001	< 0.001	0.140
J-OSDI 1	J-OSDI 0	-0.030	< 0.001	< 0.001	0.005
J-OSDI 1	J-OSDI 11	0.084	< 0.001	< 0.001	0.123
J-OSDI 1	J-OSDI 12	0.056	< 0.001	< 0.001	0.100

J-OSDI 2	J-OSDI 3	0.113	< 0.001	< 0.001	0.153
J-OSDI 2	J-OSDI 4	0.112	< 0.001	< 0.001	0.128
J-OSDI 2	J-OSDI 5	0.083	< 0.001	< 0.001	0.078
J-OSDI 2	J-OSDI 6	0.041	< 0.001	< 0.001	0.021
J-OSDI 2	J-OSDI 7	0.001	< 0.001	< 0.001	-0.010
J-OSDI 2	J-OSDI 8	0.020	< 0.001	< 0.001	0.013
J-OSDI 2	J-OSDI 9	0.018	< 0.001	< 0.001	0.029
J-OSDI 2	J-OSDI 10	-0.046	< 0.001	< 0.001	-0.049
J-OSDI 2	J-OSDI 11	0.069	< 0.001	< 0.001	0.069
J-OSDI 2	J-OSDI 12	0.022	< 0.001	< 0.001	0.019
J-OSDI 3	J-OSDI 4	0.027	< 0.001	< 0.001	0.003
J-OSDI 3	J-OSDI 5	0.032	< 0.001	< 0.001	0.003
J-OSDI 3	J-OSDI 6	0.090	< 0.001	< 0.001	0.103
J-OSDI 3	J-OSDI 7	0.001	< 0.001	< 0.001	-0.005
J-OSDI 3	J-OSDI 8	0.068	< 0.001	< 0.001	0.067
J-OSDI 3	J-OSDI 9	0.071	< 0.001	< 0.001	0.071
J-OSDI 3	J-OSDI 10	0.018	< 0.001	< 0.001	0.028
J-OSDI 3	J-OSDI 11	0.030	< 0.001	< 0.001	0.047
J-OSDI 3	J-OSDI 12	-0.004	< 0.001	< 0.001	0.017
J-OSDI 4	J-OSDI 5	0.238	< 0.001	< 0.001	0.304
J-OSDI 4	J-OSDI 6	0.112	< 0.001	< 0.001	0.134
J-OSDI 4	J-OSDI 7	0.064	< 0.001	< 0.001	0.041
J-OSDI 4	J-OSDI 8	0.022	< 0.001	< 0.001	0.012

J-OSDI 4	J-OSDI 9	0.026	< 0.001	< 0.001	0.019
J-OSDI 4	J-OSDI 10	-0.040	< 0.001	< 0.001	-0.061
J-OSDI 4	J-OSDI 11	0.098	< 0.001	< 0.001	0.108
J-OSDI 4	J-OSDI 12	0.003	< 0.001	< 0.001	-0.005
J-OSDI 5	J-OSDI 6	0.123	< 0.001	< 0.001	0.176
J-OSDI 5	J-OSDI 7	0.131	< 0.001	< 0.001	0.139
J-OSDI 5	J-OSDI 8	0.026	< 0.001	< 0.001	-0.004
J-OSDI 5	J-OSDI 9	0.057	< 0.001	< 0.001	0.085
J-OSDI 5	J-OSDI 10	-0.007	< 0.001	< 0.001	-0.013
J-OSDI 5	J-OSDI 11	0.086	< 0.001	< 0.001	0.131
J-OSDI 5	J-OSDI 12	-0.021	< 0.001	< 0.001	-0.032
J-OSDI 6	J-OSDI 7	0.064	< 0.001	< 0.001	0.052
J-OSDI 6	J-OSDI 8	0.177	< 0.001	< 0.001	0.222
J-OSDI 6	J-OSDI 9	0.120	< 0.001	< 0.001	0.132
J-OSDI 6	J-OSDI 10	0.114	< 0.001	< 0.001	0.172
J-OSDI 6	J-OSDI 11	-0.029	< 0.001	< 0.001	0.002
J-OSDI 6	J-OSDI 12	-0.111	< 0.001	< 0.001	-0.112
J-OSDI 7	J-OSDI 8	0.005	< 0.001	< 0.001	0.001
J-OSDI 7	J-OSDI 9	0.020	< 0.001	< 0.001	-0.015
J-OSDI 7	J-OSDI 10	0.034	< 0.001	< 0.001	0.024
J-OSDI 7	J-OSDI 11	0.011	< 0.001	< 0.001	0.001
J-OSDI 7	J-OSDI 12	-0.049	< 0.001	< 0.001	-0.056
J-OSDI 8	J-OSDI 9	0.191	< 0.001	< 0.001	0.240

J-OSDI 8	J-OSDI 10	0.054	< 0.001	< 0.001	-0.005
J-OSDI 8	J-OSDI 11	-0.074	< 0.001	< 0.001	-0.078
J-OSDI 8	J-OSDI 12	-0.083	< 0.001	< 0.001	-0.045
J-OSDI 9	J-OSDI 10	0.125	< 0.001	< 0.001	0.108
J-OSDI 9	J-OSDI 11	-0.101	< 0.001	< 0.001	-0.152
J-OSDI 9	J-OSDI 12	-0.159	< 0.001	< 0.001	-0.227
J-OSDI 10	J-OSDI 11	-0.187	< 0.001	< 0.001	-0.252
J-OSDI 10	J-OSDI 12	-0.253	< 0.001	< 0.001	-0.346
J-OSDI 11	J-OSDI 12	0.158	< 0.001	< 0.001	0.175

d. Cluster 3

Node 1	Node 2	wTO with positive and negative signs	*P*-value	Adjusted *P*-value	Pearson coefficient value
J-OSDI 1	J-OSDI 2	0.061	< 0.001	< 0.001	0.068
J-OSDI 1	J-OSDI 3	0.071	< 0.001	< 0.001	0.078
J-OSDI 1	J-OSDI 4	0.010	< 0.001	< 0.001	0.008
J-OSDI 1	J-OSDI 5	-0.023	< 0.001	< 0.001	-0.048
J-OSDI 1	J-OSDI 6	-0.046	< 0.001	< 0.001	-0.061
J-OSDI 1	J-OSDI 7	0.014	< 0.001	< 0.001	0.017
J-OSDI 1	J-OSDI 8	-0.025	< 0.001	< 0.001	-0.060
J-OSDI 1	J-OSDI 9	-0.025	< 0.001	< 0.001	-0.041

J-OSDI 1	J-OSDI 10	-0.073	< 0.001	< 0.001	-0.068
J-OSDI 1	J-OSDI 11	-0.118	< 0.001	< 0.001	-0.165
J-OSDI 1	J-OSDI 12	-0.121	< 0.001	< 0.001	-0.161
J-OSDI 2	J-OSDI 3	-0.045	< 0.001	< 0.001	-0.098
J-OSDI 2	J-OSDI 4	-0.011	< 0.001	< 0.001	0.015
J-OSDI 2	J-OSDI 5	-0.120	< 0.001	< 0.001	-0.200
J-OSDI 2	J-OSDI 6	-0.109	< 0.001	< 0.001	-0.153
J-OSDI 2	J-OSDI 7	-0.008	< 0.001	< 0.001	-0.016
J-OSDI 2	J-OSDI 8	-0.067	< 0.001	< 0.001	-0.109
J-OSDI 2	J-OSDI 9	-0.008	< 0.001	< 0.001	-0.025
J-OSDI 2	J-OSDI 10	-0.017	< 0.001	< 0.001	-0.063
J-OSDI 2	J-OSDI 11	-0.012	< 0.001	< 0.001	-0.023
J-OSDI 2	J-OSDI 12	-0.032	< 0.001	< 0.001	-0.057
J-OSDI 3	J-OSDI 4	0.003	< 0.001	< 0.001	0.012
J-OSDI 3	J-OSDI 5	0.004	< 0.001	< 0.001	-0.030
J-OSDI 3	J-OSDI 6	0.023	< 0.001	< 0.001	0.035
J-OSDI 3	J-OSDI 7	-0.008	< 0.001	< 0.001	0.003
J-OSDI 3	J-OSDI 8	0.059	< 0.001	< 0.001	0.065
J-OSDI 3	J-OSDI 9	0.040	< 0.001	< 0.001	0.054
J-OSDI 3	J-OSDI 10	-0.115	< 0.001	< 0.001	-0.158
J-OSDI 3	J-OSDI 11	-0.081	< 0.001	< 0.001	-0.087
J-OSDI 3	J-OSDI 12	-0.146	< 0.001	< 0.001	-0.194
J-OSDI 4	J-OSDI 5	0.142	< 0.001	< 0.001	0.175

J-OSDI 4	J-OSDI 6	0.007	< 0.001	< 0.001	0.007
J-OSDI 4	J-OSDI 7	0.086	< 0.001	< 0.001	0.103
J-OSDI 4	J-OSDI 8	-0.008	< 0.001	< 0.001	-0.006
J-OSDI 4	J-OSDI 9	-0.109	< 0.001	< 0.001	-0.123
J-OSDI 4	J-OSDI 10	-0.053	< 0.001	< 0.001	-0.050
J-OSDI 4	J-OSDI 11	-0.076	< 0.001	< 0.001	-0.080
J-OSDI 4	J-OSDI 12	0.029	< 0.001	< 0.001	0.048
J-OSDI 5	J-OSDI 6	0.038	< 0.001	< 0.001	0.024
J-OSDI 5	J-OSDI 7	0.045	< 0.001	< 0.001	0.049
J-OSDI 5	J-OSDI 8	0.029	< 0.001	< 0.001	0.028
J-OSDI 5	J-OSDI 9	-0.082	< 0.001	< 0.001	-0.093
J-OSDI 5	J-OSDI 10	-0.076	< 0.001	< 0.001	-0.122
J-OSDI 5	J-OSDI 11	-0.091	< 0.001	< 0.001	-0.127
J-OSDI 5	J-OSDI 12	0.038	< 0.001	< 0.001	0.042
J-OSDI 6	J-OSDI 7	-0.023	< 0.001	< 0.001	-0.041
J-OSDI 6	J-OSDI 8	-0.057	< 0.001	< 0.001	-0.106
J-OSDI 6	J-OSDI 9	-0.025	< 0.001	< 0.001	-0.040
J-OSDI 6	J-OSDI 10	-0.011	< 0.001	< 0.001	-0.032
J-OSDI 6	J-OSDI 11	0.022	< 0.001	< 0.001	0.031
J-OSDI 6	J-OSDI 12	0.025	< 0.001	< 0.001	0.016
J-OSDI 7	J-OSDI 8	-0.058	< 0.001	< 0.001	-0.080
J-OSDI 7	J-OSDI 9	-0.026	< 0.001	< 0.001	-0.018
J-OSDI 7	J-OSDI 10	0.041	< 0.001	< 0.001	0.064

J-OSDI 7	J-OSDI 11	-0.006	< 0.001	< 0.001	-0.007
J-OSDI 7	J-OSDI 12	0.011	< 0.001	< 0.001	0.001
J-OSDI 8	J-OSDI 9	0.017	< 0.001	< 0.001	0.011
J-OSDI 8	J-OSDI 10	-0.031	< 0.001	< 0.001	-0.033
J-OSDI 8	J-OSDI 11	-0.021	< 0.001	< 0.001	-0.024
J-OSDI 8	J-OSDI 12	-0.060	< 0.001	< 0.001	-0.091
J-OSDI 9	J-OSDI 10	0.003	< 0.001	< 0.001	-0.011
J-OSDI 9	J-OSDI 11	0.018	< 0.001	< 0.001	0.005
J-OSDI 9	J-OSDI 12	-0.010	< 0.001	< 0.001	0.001
J-OSDI 10	J-OSDI 11	0.188	< 0.001	< 0.001	0.250
J-OSDI 10	J-OSDI 12	0.065	< 0.001	< 0.001	0.059
J-OSDI 11	J-OSDI 12	0.050	< 0.001	< 0.001	0.030

e. Cluster 4

Node 1	Node 2	wTO with positive and negative signs	*P*-value	Adjusted *P*-value	Pearson coefficient value
J-OSDI 1	J-OSDI 2	0.180	< 0.001	< 0.001	0.152
J-OSDI 1	J-OSDI 3	0.188	< 0.001	< 0.001	0.161
J-OSDI 1	J-OSDI 4	0.281	< 0.001	< 0.001	0.252
J-OSDI 1	J-OSDI 5	0.259	0.008	0.009	0.242
J-OSDI 1	J-OSDI 6	0.103	0.008	0.009	-0.005

J-OSDI 1	J-OSDI 7	-0.155	< 0.001	< 0.001	-0.072
J-OSDI 1	J-OSDI 8	0.140	< 0.001	< 0.001	0.060
J-OSDI 1	J-OSDI 9	0.089	0.004	0.005	0.027
J-OSDI 1	J-OSDI 0	0.121	< 0.001	< 0.001	0.122
J-OSDI 1	J-OSDI 11	0.069	< 0.001	< 0.001	0.031
J-OSDI 1	J-OSDI 12	0.040	< 0.001	< 0.001	0.027
J-OSDI 2	J-OSDI 3	0.268	< 0.001	< 0.001	0.405
J-OSDI 2	J-OSDI 4	0.236	0.004	0.005	0.290
J-OSDI 2	J-OSDI 5	0.196	0.004	0.005	0.142
J-OSDI 2	J-OSDI 6	0.051	< 0.001	< 0.001	-0.087
J-OSDI 2	J-OSDI 7	-0.145	< 0.001	< 0.001	-0.103
J-OSDI 2	J-OSDI 8	0.112	< 0.001	< 0.001	0.012
J-OSDI 2	J-OSDI 9	0.107	0.008	0.009	0.044
J-OSDI 2	J-OSDI 10	0.141	0.004	0.005	0.174
J-OSDI 2	J-OSDI 11	0.079	< 0.001	< 0.001	0.058
J-OSDI 2	J-OSDI 12	0.066	< 0.001	< 0.001	0.063
J-OSDI 3	J-OSDI 4	0.244	< 0.001	< 0.001	0.149
J-OSDI 3	J-OSDI 5	0.252	< 0.001	< 0.001	0.276
J-OSDI 3	J-OSDI 6	0.095	< 0.001	< 0.001	0.004
J-OSDI 3	J-OSDI 7	-0.175	< 0.001	< 0.001	-0.181
J-OSDI 3	J-OSDI 8	0.157	< 0.001	< 0.001	0.148
J-OSDI 3	J-OSDI 9	0.208	0.004	0.005	0.241
J-OSDI 3	J-OSDI 10	0.130	0.004	0.005	0.139

J-OSDI 3	J-OSDI 11	0.069	0.004	0.005	0.033
J-OSDI 3	J-OSDI 12	0.052	< 0.001	< 0.001	0.009
J-OSDI 4	J-OSDI 5	0.345	< 0.001	< 0.001	0.595
J-OSDI 4	J-OSDI 6	0.321	0.004	0.005	0.333
J-OSDI 4	J-OSDI 7	-0.300	< 0.001	< 0.001	-0.345
J-OSDI 4	J-OSDI 8	0.288	< 0.001	0.000	0.293
J-OSDI 4	J-OSDI 9	0.191	0.024	0.024	0.110
J-OSDI 4	J-OSDI 10	0.187	0.004	0.005	0.174
J-OSDI 4	J-OSDI 11	0.183	0.004	0.005	0.153
J-OSDI 4	J-OSDI 12	0.103	0.016	0.017	0.029
J-OSDI 5	J-OSDI 6	0.314	< 0.001	< 0.001	0.386
J-OSDI 5	J-OSDI 7	-0.252	< 0.001	< 0.001	-0.248
J-OSDI 5	J-OSDI 8	0.244	< 0.001	< 0.001	0.234
J-OSDI 5	J-OSDI 9	0.302	0.028	0.028	0.343
J-OSDI 5	J-OSDI 10	0.110	0.004	0.005	-0.031
J-OSDI 5	J-OSDI 11	0.130	0.004	0.005	0.111
J-OSDI 5	J-OSDI 12	0.041	0.004	0.005	-0.078
J-OSDI 6	J-OSDI 7	-0.292	< 0.001	< 0.001	-0.302
J-OSDI 6	J-OSDI 8	0.322	< 0.001	< 0.001	0.409
J-OSDI 6	J-OSDI 9	0.097	0.004	0.005	0.039
J-OSDI 6	J-OSDI 10	0.117	< 0.001	< 0.001	0.057
J-OSDI 6	J-OSDI 11	0.158	0.004	0.005	0.153
J-OSDI 6	J-OSDI 12	0.099	0.004	0.005	0.050

J-OSDI 7	J-OSDI 8	-0.320	< 0.001	< 0.001	-0.470
J-OSDI 7	J-OSDI 9	-0.070	0.012	0.013	0.035
J-OSDI 7	J-OSDI 10	-0.212	0.004	0.005	-0.198
J-OSDI 7	J-OSDI 11	-0.187	0.004	0.005	-0.163
J-OSDI 7	J-OSDI 12	-0.158	0.008	0.009	-0.111
J-OSDI 8	J-OSDI 9	0.073	0.012	0.013	0.016
J-OSDI 8	J-OSDI 10	0.239	0.004	0.005	0.304
J-OSDI 8	J-OSDI 11	0.178	0.004	0.005	0.076
J-OSDI 8	J-OSDI 12	0.181	0.012	0.013	0.195
J-OSDI 9	J-OSDI 10	-0.006	< 0.001	< 0.001	-0.059
J-OSDI 9	J-OSDI 11	0.002	< 0.001	< 0.001	-0.057
J-OSDI 9	J-OSDI 12	-0.029	0.004	0.005	-0.004
J-OSDI 10	J-OSDI 11	0.133	< 0.001	< 0.001	0.097
J-OSDI 10	J-OSDI 12	0.190	< 0.001	< 0.001	0.228
J-OSDI 11	J-OSDI 12	0.261	< 0.001	< 0.001	0.404

f. Cluster 5

Node 1	Node 2	wTO with positive and negative signs	*P*-value	Adjusted *P*-value	Pearson coefficient value
J-OSDI 1	J-OSDI 2	0.096	< 0.001	< 0.001	0.105
J-OSDI 1	J-OSDI 3	0.059	< 0.001	< 0.001	0.048

J-OSDI 1	J-OSDI 4	0.136	< 0.001	< 0.001	0.121
J-OSDI 1	J-OSDI 5	0.138	< 0.001	< 0.001	0.135
J-OSDI 1	J-OSDI 6	0.061	< 0.001	< 0.001	0.039
J-OSDI 1	J-OSDI 7	0.029	< 0.001	< 0.001	0.073
J-OSDI 1	J-OSDI 8	0.035	< 0.001	< 0.001	0.031
J-OSDI 1	J-OSDI 9	0.026	< 0.001	< 0.001	-0.039
J-OSDI 1	J-OSDI 0	0.010	< 0.001	< 0.001	0.024
J-OSDI 1	J-OSDI 11	0.147	< 0.001	< 0.001	0.151
J-OSDI 1	J-OSDI 12	0.050	< 0.001	< 0.001	-0.003
J-OSDI 2	J-OSDI 3	0.275	< 0.001	< 0.001	0.352
J-OSDI 2	J-OSDI 4	0.061	< 0.001	< 0.001	0.086
J-OSDI 2	J-OSDI 5	0.034	< 0.001	< 0.001	-0.035
J-OSDI 2	J-OSDI 6	0.050	< 0.001	< 0.001	0.019
J-OSDI 2	J-OSDI 7	-0.056	< 0.001	< 0.001	-0.053
J-OSDI 2	J-OSDI 8	0.071	< 0.001	< 0.001	0.072
J-OSDI 2	J-OSDI 9	0.035	< 0.001	< 0.001	0.017
J-OSDI 2	J-OSDI 10	-0.117	< 0.001	< 0.001	-0.166
J-OSDI 2	J-OSDI 11	0.182	< 0.001	< 0.001	0.246
J-OSDI 2	J-OSDI 12	0.067	< 0.001	< 0.001	0.034
J-OSDI 3	J-OSDI 4	0.028	< 0.001	< 0.001	0.005
J-OSDI 3	J-OSDI 5	-0.003	< 0.001	< 0.001	-0.047
J-OSDI 3	J-OSDI 6	0.034	< 0.001	< 0.001	0.027
J-OSDI 3	J-OSDI 7	-0.083	< 0.001	< 0.001	-0.105

J-OSDI 3	J-OSDI 8	0.055	< 0.001	< 0.001	0.048
J-OSDI 3	J-OSDI 9	0.019	< 0.001	< 0.001	-0.011
J-OSDI 3	J-OSDI 10	-0.058	< 0.001	< 0.001	-0.038
J-OSDI 3	J-OSDI 11	0.158	< 0.001	< 0.001	0.179
J-OSDI 3	J-OSDI 12	0.027	< 0.001	< 0.001	-0.016
J-OSDI 4	J-OSDI 5	0.344	< 0.001	< 0.001	0.518
J-OSDI 4	J-OSDI 6	0.141	< 0.001	< 0.001	0.147
J-OSDI 4	J-OSDI 7	-0.043	< 0.001	< 0.001	-0.055
J-OSDI 4	J-OSDI 8	0.031	< 0.001	< 0.001	-0.003
J-OSDI 4	J-OSDI 9	0.138	< 0.001	< 0.001	0.134
J-OSDI 4	J-OSDI 10	0.038	< 0.001	< 0.001	0.040
J-OSDI 4	J-OSDI 11	0.193	< 0.001	< 0.001	0.170
J-OSDI 4	J-OSDI 12	0.107	< 0.001	< 0.001	0.053
J-OSDI 5	J-OSDI 6	0.138	< 0.001	< 0.001	0.145
J-OSDI 5	J-OSDI 7	-0.028	< 0.001	< 0.001	-0.028
J-OSDI 5	J-OSDI 8	0.019	< 0.001	< 0.001	-0.074
J-OSDI 5	J-OSDI 9	0.143	< 0.001	< 0.001	0.148
J-OSDI 5	J-OSDI 10	0.051	< 0.001	< 0.001	0.022
J-OSDI 5	J-OSDI 11	0.193	< 0.001	< 0.001	0.256
J-OSDI 5	J-OSDI 12	0.149	< 0.001	< 0.001	0.125
J-OSDI 6	J-OSDI 7	0.028	< 0.001	< 0.001	0.037
J-OSDI 6	J-OSDI 8	0.196	< 0.001	< 0.001	0.281
J-OSDI 6	J-OSDI 9	0.163	< 0.001	< 0.001	0.177

J-OSDI 6	J-OSDI 10	-0.009	< 0.001	< 0.001	-0.036
J-OSDI 6	J-OSDI 11	0.160	< 0.001	< 0.001	0.145
J-OSDI 6	J-OSDI 12	0.083	< 0.001	< 0.001	0.046
J-OSDI 7	J-OSDI 8	0.058	< 0.001	< 0.001	0.090
J-OSDI 7	J-OSDI 9	0.002	< 0.001	< 0.001	-0.003
J-OSDI 7	J-OSDI 10	-0.010	< 0.001	< 0.001	-0.018
J-OSDI 7	J-OSDI 11	-0.031	< 0.001	< 0.001	-0.070
J-OSDI 7	J-OSDI 12	0.052	< 0.001	< 0.001	0.109
J-OSDI 8	J-OSDI 9	0.168	< 0.001	< 0.001	0.235
J-OSDI 8	J-OSDI 10	-0.012	< 0.001	< 0.001	-0.021
J-OSDI 8	J-OSDI 11	0.131	< 0.001	< 0.001	0.143
J-OSDI 8	J-OSDI 12	0.062	< 0.001	< 0.001	0.028
J-OSDI 9	J-OSDI 10	0.067	< 0.001	< 0.001	0.091
J-OSDI 9	J-OSDI 11	0.184	< 0.001	< 0.001	0.204
J-OSDI 9	J-OSDI 12	0.104	< 0.001	< 0.001	0.070
J-OSDI 10	J-OSDI 11	0.024	< 0.001	< 0.001	0.060
J-OSDI 10	J-OSDI 12	0.009	< 0.001	< 0.001	-0.012
J-OSDI 11	J-OSDI 12	0.300	0.004	0.004	0.387

g. Cluster 6

Node 1	Node 2	wTO with positive and negative signs	*P*-value	Adjusted *P*-value	Pearson coefficient value
J-OSDI 1	J-OSDI 2	-0.100	< 0.001	< 0.001	-0.132
J-OSDI 1	J-OSDI 3	-0.070	< 0.001	< 0.001	-0.078
J-OSDI 1	J-OSDI 4	0.044	< 0.001	< 0.001	0.080
J-OSDI 1	J-OSDI 5	0.062	< 0.001	< 0.001	0.086
J-OSDI 1	J-OSDI 6	-0.026	< 0.001	< 0.001	-0.052
J-OSDI 1	J-OSDI 7	-0.043	< 0.001	< 0.001	-0.074
J-OSDI 1	J-OSDI 8	-0.039	< 0.001	< 0.001	-0.067
J-OSDI 1	J-OSDI 9	0.041	< 0.001	< 0.001	0.101
J-OSDI 1	J-OSDI 0	0.002	< 0.001	< 0.001	0.005
J-OSDI 1	J-OSDI 11	-0.032	< 0.001	< 0.001	0.013
J-OSDI 1	J-OSDI 12	-0.151	< 0.001	< 0.001	-0.209
J-OSDI 2	J-OSDI 3	0.195	< 0.001	< 0.001	0.284
J-OSDI 2	J-OSDI 4	0.089	< 0.001	< 0.001	0.089
J-OSDI 2	J-OSDI 5	0.059	< 0.001	< 0.001	0.072
J-OSDI 2	J-OSDI 6	0.082	< 0.001	< 0.001	0.106
J-OSDI 2	J-OSDI 7	-0.054	< 0.001	< 0.001	-0.053
J-OSDI 2	J-OSDI 8	0.010	< 0.001	< 0.001	-0.080
J-OSDI 2	J-OSDI 9	0.022	< 0.001	< 0.001	0.002
J-OSDI 2	J-OSDI 10	0.021	< 0.001	< 0.001	0.020
J-OSDI 2	J-OSDI 11	0.226	< 0.001	< 0.001	0.295

J-OSDI 2	J-OSDI 12	0.167	< 0.001	< 0.001	0.211
J-OSDI 3	J-OSDI 4	0.110	< 0.001	< 0.001	0.137
J-OSDI 3	J-OSDI 5	0.066	< 0.001	< 0.001	0.047
J-OSDI 3	J-OSDI 6	0.107	< 0.001	< 0.001	0.120
J-OSDI 3	J-OSDI 7	-0.041	< 0.001	< 0.001	-0.053
J-OSDI 3	J-OSDI 8	0.079	< 0.001	< 0.001	0.125
J-OSDI 3	J-OSDI 9	0.070	< 0.001	< 0.001	0.067
J-OSDI 3	J-OSDI 10	0.009	< 0.001	< 0.001	-0.048
J-OSDI 3	J-OSDI 11	0.116	< 0.001	< 0.001	0.102
J-OSDI 3	J-OSDI 12	0.120	< 0.001	< 0.001	0.118
J-OSDI 4	J-OSDI 5	0.196	< 0.001	< 0.001	0.267
J-OSDI 4	J-OSDI 6	0.141	< 0.001	< 0.001	0.164
J-OSDI 4	J-OSDI 7	-0.002	< 0.001	< 0.001	-0.003
J-OSDI 4	J-OSDI 8	0.089	< 0.001	< 0.001	0.086
J-OSDI 4	J-OSDI 9	0.135	< 0.001	< 0.001	0.140
J-OSDI 4	J-OSDI 10	0.060	< 0.001	< 0.001	0.043
J-OSDI 4	J-OSDI 11	0.087	< 0.001	< 0.001	0.096
J-OSDI 4	J-OSDI 12	0.027	< 0.001	< 0.001	0.011
J-OSDI 5	J-OSDI 6	0.115	< 0.001	< 0.001	0.115
J-OSDI 5	J-OSDI 7	0.010	< 0.001	< 0.001	0.017
J-OSDI 5	J-OSDI 8	0.072	< 0.001	< 0.001	0.082
J-OSDI 5	J-OSDI 9	0.124	< 0.001	< 0.001	0.135
J-OSDI 5	J-OSDI 10	0.041	< 0.001	< 0.001	0.004

J-OSDI 5	J-OSDI 11	0.066	< 0.001	< 0.001	0.093
J-OSDI 5	J-OSDI 12	-0.017	< 0.001	< 0.001	-0.083
J-OSDI 6	J-OSDI 7	0.049	< 0.001	< 0.001	0.060
J-OSDI 6	J-OSDI 8	0.140	< 0.001	< 0.001	0.153
J-OSDI 6	J-OSDI 9	0.198	< 0.001	< 0.001	0.282
J-OSDI 6	J-OSDI 10	0.101	< 0.001	< 0.001	0.084
J-OSDI 6	J-OSDI 11	0.046	< 0.001	< 0.001	0.017
J-OSDI 6	J-OSDI 12	0.060	< 0.001	< 0.001	0.044
J-OSDI 7	J-OSDI 8	0.060	< 0.001	< 0.001	0.062
J-OSDI 7	J-OSDI 9	0.030	< 0.001	< 0.001	0.012
J-OSDI 7	J-OSDI 10	0.055	< 0.001	< 0.001	0.065
J-OSDI 7	J-OSDI 11	-0.051	< 0.001	< 0.001	-0.052
J-OSDI 7	J-OSDI 12	-0.026	< 0.001	< 0.001	-0.037
J-OSDI 8	J-OSDI 9	0.184	< 0.001	< 0.001	0.257
J-OSDI 8	J-OSDI 10	0.153	< 0.001	< 0.001	0.183
J-OSDI 8	J-OSDI 11	0.015	< 0.001	< 0.001	-0.021
J-OSDI 8	J-OSDI 12	0.091	< 0.001	< 0.001	0.159
J-OSDI 9	J-OSDI 10	0.146	< 0.001	< 0.001	0.156
J-OSDI 9	J-OSDI 11	0.022	< 0.001	< 0.001	-0.019
J-OSDI 9	J-OSDI 12	0.041	< 0.001	< 0.001	0.057
J-OSDI 10	J-OSDI 11	0.066	< 0.001	< 0.001	0.108
J-OSDI 10	J-OSDI 12	0.043	< 0.001	< 0.001	0.014
J-OSDI 11	J-OSDI 12	0.154	< 0.001	< 0.001	0.214

h. Cluster 7

Node 1	Node 2	wTO with positive and negative signs	*P*-value	Adjusted *P*-value	Pearson coefficient value
J-OSDI 1	J-OSDI 2	0.117	< 0.001	< 0.001	0.114
J-OSDI 1	J-OSDI 3	0.082	< 0.001	< 0.001	0.065
J-OSDI 1	J-OSDI 4	0.185	< 0.001	< 0.001	0.183
J-OSDI 1	J-OSDI 5	0.211	< 0.001	< 0.001	0.214
J-OSDI 1	J-OSDI 6	-0.096	< 0.001	< 0.001	-0.104
J-OSDI 1	J-OSDI 7	-0.158	< 0.001	< 0.001	-0.176
J-OSDI 1	J-OSDI 8	-0.016	< 0.001	< 0.001	0.034
J-OSDI 1	J-OSDI 9	0.033	< 0.001	< 0.001	0.040
J-OSDI 1	J-OSDI 0	0.038	< 0.001	< 0.001	0.048
J-OSDI 1	J-OSDI 11	-0.038	< 0.001	< 0.001	-0.039
J-OSDI 1	J-OSDI 12	0.001	< 0.001	< 0.001	0.016
J-OSDI 2	J-OSDI 3	0.226	< 0.001	< 0.001	0.318
J-OSDI 2	J-OSDI 4	0.168	< 0.001	< 0.001	0.195
J-OSDI 2	J-OSDI 5	0.145	< 0.001	< 0.001	0.168
J-OSDI 2	J-OSDI 6	-0.031	< 0.001	< 0.001	-0.036
J-OSDI 2	J-OSDI 7	-0.110	< 0.001	< 0.001	-0.083
J-OSDI 2	J-OSDI 8	-0.042	< 0.001	< 0.001	-0.035

J-OSDI 2	J-OSDI 9	-0.012	< 0.001	< 0.001	-0.043
J-OSDI 2	J-OSDI 10	0.109	< 0.001	< 0.001	0.127
J-OSDI 2	J-OSDI 11	0.048	< 0.001	< 0.001	0.105
J-OSDI 2	J-OSDI 12	0.063	< 0.001	< 0.001	0.081
J-OSDI 3	J-OSDI 4	0.145	< 0.001	< 0.001	0.161
J-OSDI 3	J-OSDI 5	0.110	< 0.001	< 0.001	0.097
J-OSDI 3	J-OSDI 6	0.038	< 0.001	< 0.001	0.132
J-OSDI 3	J-OSDI 7	-0.100	< 0.001	< 0.001	-0.109
J-OSDI 3	J-OSDI 8	0.004	< 0.001	< 0.001	0.036
J-OSDI 3	J-OSDI 9	-0.016	< 0.001	< 0.001	0.001
J-OSDI 3	J-OSDI 10	0.088	< 0.001	< 0.001	0.096
J-OSDI 3	J-OSDI 11	0.019	< 0.001	< 0.001	0.025
J-OSDI 3	J-OSDI 12	0.025	< 0.001	< 0.001	0.010
J-OSDI 4	J-OSDI 5	0.267	< 0.001	< 0.001	0.434
J-OSDI 4	J-OSDI 6	-0.104	< 0.001	< 0.001	-0.096
J-OSDI 4	J-OSDI 7	-0.183	< 0.001	< 0.001	-0.164
J-OSDI 4	J-OSDI 8	-0.141	< 0.001	< 0.001	-0.146
J-OSDI 4	J-OSDI 9	-0.069	< 0.001	< 0.001	-0.158
J-OSDI 4	J-OSDI 10	0.001	< 0.001	< 0.001	0.014
J-OSDI 4	J-OSDI 11	-0.109	< 0.001	< 0.001	-0.140
J-OSDI 4	J-OSDI 12	-0.051	< 0.001	< 0.001	-0.026
J-OSDI 5	J-OSDI 6	-0.191	< 0.001	< 0.001	-0.235
J-OSDI 5	J-OSDI 7	-0.216	< 0.001	< 0.001	-0.213

J-OSDI 5	J-OSDI 8	-0.137	< 0.001	< 0.001	-0.153
J-OSDI 5	J-OSDI 9	0.027	< 0.001	< 0.001	0.095
J-OSDI 5	J-OSDI 10	-0.008	< 0.001	< 0.001	-0.018
J-OSDI 5	J-OSDI 11	-0.117	< 0.001	< 0.001	-0.154
J-OSDI 5	J-OSDI 12	-0.078	< 0.001	< 0.001	-0.089
J-OSDI 6	J-OSDI 7	0.088	< 0.001	< 0.001	0.083
J-OSDI 6	J-OSDI 8	0.075	< 0.001	< 0.001	0.098
J-OSDI 6	J-OSDI 9	-0.113	< 0.001	< 0.001	-0.195
J-OSDI 6	J-OSDI 10	0.012	< 0.001	< 0.001	0.033
J-OSDI 6	J-OSDI 11	0.023	< 0.001	< 0.001	0.008
J-OSDI 6	J-OSDI 12	0.010	< 0.001	< 0.001	-0.015
J-OSDI 7	J-OSDI 8	0.032	< 0.001	< 0.001	0.005
J-OSDI 7	J-OSDI 9	-0.033	< 0.001	< 0.001	-0.047
J-OSDI 7	J-OSDI 10	-0.029	< 0.001	< 0.001	-0.031
J-OSDI 7	J-OSDI 11	0.034	< 0.001	< 0.001	0.020
J-OSDI 7	J-OSDI 12	0.021	< 0.001	< 0.001	0.025
J-OSDI 8	J-OSDI 9	0.071	< 0.001	< 0.001	0.122
J-OSDI 8	J-OSDI 10	0.046	< 0.001	< 0.001	0.060
J-OSDI 8	J-OSDI 11	0.022	< 0.001	< 0.001	-0.037
J-OSDI 8	J-OSDI 12	0.051	< 0.001	< 0.001	0.065
J-OSDI 9	J-OSDI 10	0.063	< 0.001	< 0.001	0.097
J-OSDI 9	J-OSDI 11	0.057	< 0.001	< 0.001	0.087
J-OSDI 9	J-OSDI 12	0.033	< 0.001	< 0.001	0.021

J-OSDI 10	J-OSDI 11	0.144	< 0.001	< 0.001	0.189
J-OSDI 10	J-OSDI 12	0.137	< 0.001	< 0.001	0.156
J-OSDI 11	J-OSDI 12	0.170	< 0.001	< 0.001	0.201

J-OSDI: Japanese version of the ocular surface disease index; wTO: weighted topological overlap.

Supplementary Table 4. Odds ratios for each cluster in symptomatic DE compared with non-symptomatic DE.

Risk factors	Cluster 1	Cluster 2	Cluster 3	Cluster 4	Cluster 5	Cluster 6	Cluster 7
Age	0.995 (0.983–1.001)	***0.971 (0.959–0.984)	1.003 (0.991–1.015)	**1.023 (1.008–1.040)	***0.950 (0.935–0.965)	0.987 (0.974–1.000)	*1.013 (1.002–1.024)
Sex	***2.39 (1.76–3.25)	***2.42 (1.84–3.18)	**1.63 (1.23–2.17)	**1.88 (1.25–2.81)	***3.32 (2.38–4.64)	***1.75 (1.30–2.37)	**1.46 (1.12–1.90)
Body mass index	1.01 (0.98–1.05)	0.99 (0.95–1.02)	0.99 (0.95–1.02)	0.97 (0.92–1.02)	0.99 (0.95–1.04)	1.03 (0.99–1.07)	1.00 (0.97–1.04)
Medical history							
Hypertension	0.94 (0.45–1.99)	1.04 (0.48–2.25)	0.86 (0.42–1.75)	1.27 (0.58–2.77)	0.51 (0.14–1.77)	1.54 (0.80–2.96)	1.28 (0.73–2.25)
Diabetes	1.11 (0.39–3.15)	1.39 (0.46–4.21)	1.23 (0.48–3.21)	1.05 (0.38–2.95)	1.94 (0.38–9.96)	0.77 (0.26–2.31)	0.71 (0.29–1.71)
Blood disease	1.52 (0.53–4.37)	1.57 (0.47–5.31)	0.19 (0.02–1.53)	0.37 (0.04–3.15)	0.34 (0.027–4.23)	0.40 (0.08–2.16)	0.95 (0.32–2.82)
Brain disease	1.13	0.99	1.49	1.11	0.96	2.13	0.14

	(0.29–4.47)	(0.22–4.42)	(0.41–5.46)	(0.20–6.09)	(0.078–11.8)	(0.63–7.22)	(0.02–1.25)
Collagen disease	3.60<br>(0.98–13.3)	2.37<br>(0.54–10.5)	0.44<br>(0.05–4.25)	0.74<br>(0.08–7.25)	*6.99<br>(1.46–33.5)	1.70<br>(0.37–7.73)	1.75<br>(0.45–6.80)
Heart disease	1.36<br>(0.51–3.61)	1.45<br>(0.49–4.24)	0.36<br>(0.08–1.68)	2.24<br>(0.73–6.84)	2.96<br>(0.97–9.02)	1.02<br>(0.35–2.96)	1.83<br>(0.82–4.08)
Kidney disease	0.87<br>(027–2.80)	2.34<br>(0.94–5.83)	1.02<br>(0.30–3.49)	0.90<br>(0.22–3.62)	1.47<br>(0.40–5.36)	0.94<br>(0.30–3.01)	1.22<br>(0.49–3.05)
Liver disease	0.99<br>(0.32–3.08)	0.24<br>(0.04–1.49)	1.02<br>(0.30–3.49)	1.33<br>(0.40–4.42)	0.99<br>(0.20–4.98)	1.88<br>(0.70–5.08)	1.02<br>(0.39–2.67)
Malignant tumor	1.37<br>(0.36–5.17)	3.08<br>(0.96–9.83)	–	0.49<br>(0.05–4.52)	–	1.08<br>(0.25–4.59)	1.00<br>(0.29–3.38)
Respiratory disease	0.83<br>(0.50–1.40)	1.20<br>(0.76–1.89)	1.07<br>(0.66–1.75)	0.85<br>(0.42–1.69)	0.98<br>(0.55–1.73)	0.89<br>(0.52–1.51)	1.15<br>(0.72–1.83)
Hay fever	**1.53<br>(1.18–1.99)	*1.33<br>(1.05–1.69)	1.25<br>(0.97–1.60)	1.08<br>(0.76–1.53)	***1.69<br>(1.28–2.22)	*1.40<br>(1.08–1.81)	1.26<br>(1.00–1.59)
Mental illness							

Depression	*1.97 (1.09–3.55)	1.59 (0.84–3.01)	0.87 (0.44–1.73)	1.67 (0.81–3.45)	1.15 (0.54–2.46)	*1.89 (1.03–3.46)	0.90 (0.47–1.72)
Schizophrenia	1.16 (0.33–4.04)	0.34 (0.07–1.78)	0.98 (0.28–3.43)	1.01 (0.20–5.22)	0.85 (0.20–3.62)	0.24 (0.03–1.97)	0.96 (0.27–3.33)
Other mental disease	1.51 (0.85–2.67)	0.89 (0.47–1.67)	1.59 (0.89–2.84)	1.11 (0.52–2.37)	1.17 (0.60–2.30)	1.28 (0.69–2.36)	1.39 (0.80–2.42)
Ophthalmic surgery							
Cataract surgery	0.55 (0.10–3.06)	0.49 (0.06–4.29)	0.53 (0.11–2.67)	0.26 (0.03–2.51)	–	0.45 (0.05–3.93)	0.67 (0.18–2.53)
LASIK	0.56 (0.18–1.80)	0.81 (0.31–2.14)	0.81 (0.31–2.14)	1.38 (0.52–3.67)	0.94 (0.31–2.82)	0.91 (0.32–2.58)	0.75 (0.28–2.00)
Other ophthalmic surgery	2.03 (1.00–4.13)	0.95 (0.43–2.09)	**2.48 (1.27–4.85)	0.89 (0.28–2.78)	0.89 (0.32–2.52)	1.22 (0.55–2.76)	0.91 (0.42–1.97)
Lifestyle habits							

Coffee (every 1 cup per day increase)	1.09 (0.99–1.20)	1.11 (1.00–1.22)	1.02 (0.92–1.13)	1.10 (0.97–1.25)	1.11 (00.99–1.25)	1.08 (0.97–1.20)	1.08 (0.99–1.19)
Contact lens use							
No	1 (reference)	1 (reference)	1 (reference)	1 (reference)	1 (reference)	1 (reference)	1 (reference)
Current use	**1.49 (1.11–1.99)	***1.66 (1.28–2.15)	1.30 (0.99–1.71)	0.71 (0.46–1.09)	***1.99 (1.49–2.68)	1.05 (0.78–1.40	0.92 (0.71–1.20)
Past use	1.10 (0.73–1.68)	*1.49 (1.01–2.20)	0.82 (0.54–1.25)	1.22 (0.75–1.99)	**1.88 (1.18–3.00)	0.84 (0.55–1.29)	**0.53 (0.35–0.80)
Screen exposure (hours)*							
<4	1 (reference)	1 (reference)	1 (reference)	1 (reference)	1 (reference)	1 (reference)	1 (reference)
4–8	1.00 (0.70–1.43)	1.18 (0.85–1.63)	1.12 (0.80–1.58)	0.88 (0.55–1.41)	1.11 (0.77–1.60)	1.35 (0.94–1.93)	1.01 (0.74–1.39)
>8	*1.50 (1.01–2.22)	1.37 (0.96–1.98)	*1.57 (1.08–2.29)	1.08 (0.64–1.84)	1.16 (0.77–1.76)	1.33 (0.88–2.01)	1.37 (0.96–1.95)

Periodic exercise (Yes *vs*. No)	0.92 (0.70–1.20)	1.24 (0.96–1.60)	0.94 (0.72–1.21)	**0.61 (0.43–0.88)	1.00 (0.75–1.33)	1.06 (0.80–1.39)	1.11 (0.86–1.42)
Sleeping time (every 1 hour per day increase)							
<6	1.24 (0.93–1.66)	0.90 (0.69–1.19)	1.05 (0.79–1.40)	0.84 (0.56–1.27)	0.84 (0.61–1.14)	0.76 (0.56–1.03)	**0.67 (0.51–0.89)
6–9	1 (reference)	1 (reference)	1 (reference)	1 (reference)	1 (reference)	1 (reference)	1 (reference)
>9	1.08 (0.75–1.55)	0.91 (0.66–1.25)	1.22 (0.88–1.69)	1.04 (0.65–1.67)	0.74 (0.51–1.08)	0.93 (0.66–1.31)	0.76 (0.54–1.05)
Smoking (Yes *vs*. No)	***1.81 (1.32–2.47)	*1.42 (1.05–1.93)	1.15 (0.84–1.57)	1.64 (1.09–2.45)	***2.21 (1.55–3.15)	*1.49 (1.08–2.06)	1.19 (0.90–1.59)
Water intake (every 100 mL per day)	1.00 (0.97–1.03)	0.99 (0.97–1.02)	1.01 (0.98–1.03)	0.99 (0.95–1.03)	1.02 (0.99–1.05)	0.98 (0.96–1.01)	1.00 (0.97–1.03)
SDS >40	***2.87	***1.73	***1.83	***2.92	***2.22	***1.91	***2.06

	(2.13–3.86)	(1.35–2.23)	(1.41–2.39)	(1.95–4.38)	(1.64–3.00)	(1.44–2.53)	(1.60–2.64)

DE: dry eye; LASIK: laser-assisted *in situ* keratomileusis; SDS: Zung Self-rating Depression Scale.

*P < 0.05, **P < 0.001, ***P < 0.001

Supplementary Table 5. Odds ratios for each cluster compared with other clusters.

Risk factors	Cluster 0 (non-symptomatic DE)	Cluster 1	Cluster 2	Cluster 3	Cluster 4	Cluster 5	Cluster 6	Cluster 7
Age	1.005 (0.999–1.011)	1.003 (0.993–1.013)	***0.978 (0.968–0.988)	1.007 (0.997–1.018)	***1.030 (1.015–1.044)	***0.960 (0.947–0.972)	0.993 (0.982–1.003	***1.022 (1.012–1.031)
Sex	***0.67 (0.57–0.78)	**1.50 (1.16–1.94)	***1.53 (1.22–1.94)	0.99 (0.78–1.26)	1.10 (0.77–1.57)	***2.13 (1.60–2.83)	1.03 (0.80–1.33)	0.85 (0.68–1.07)
Body mass index	1.01 (0.99–1.03)	1.02 (0.99–1.04)	0.98 (0.95–1.01)	0.99 (0.96–1.02)	0.97 (0.93–1.02)	0.99 (0.96–1.02)	*1.03 (1.001–1.062)	1.00 (0.97–1.03)
Medical history								

Hypertension	0.79 (0.54–1.14)	0.77 (0.41–1.43)	1.08 (0.56–2.09)	0.83 (0.45–1.53)	1.12 (0.57–2.18)	0.85 (0.32–2.28)	1.50 (0.85–2.62)	1.01 (0.62–1.62)
Diabetes	0.92 (0.54–1.58)	0.77 (0.31–1.92)	1.17 (0.44–3.08)	1.36 (0.61–3.03)	1.27 (0.57–2.18)	1.30 (0.37–4.61)	0.60 (0.23–1.59)	0.74 (0.35–1.54)
Blood disease	1.98 (0.99–3.96)	2.06 (0.35–3.37)	1.62 (0.59–4.39)	0.23 (0.03–1.70)	0.34 (0.04–2.61)	0.35 (0.05–2.71)	0.45 (0.10–1.97)	1.17 (0.46–2.96)
Brain disease	1.19 (0.56–2.55)	1.09 (0.35–3.37)	0.99 (0.29–3.40)	1.40 (0.47–4.16)	0.96 (0.21–4.35)	0.46 (0.06–3.53)	2.33 (0.85–6.34)	0.18 (0.02–1.33)
Collagen disease	0.90 (0.43–1.02)	1.98 (0.81–4.83)	1.10 (0.37–3.29)	0.28 (0.04–2.13)	0.43 (0.06–3.32)	*3.22 (1.22–8.51)	1.01 (0.30–3.48)	1.10 (0.40–3.03)
Heart disease	0.89	1.11	0.83	*0.22	1.55	1.70	0.79	1.64

	(0.52–1.51)	(0.52–2.37)	(0.36–1.89)	(0.05–0.93)	(0.62–3.87)	(0.78–3.69)	(0.33–1.90)	(0.88–3.06)
Kidney disease	0.68 (0.39–1.19)	0.72 (0.30–1.74)	1.63 (0.81–3.24)	*2.03 (1.02–4.02)	0.71 (0.21–2.37)	1.02 (0.42–2.52)	0.72 (0.28–1.87)	0.95 (0.45–2.00)
Liver disease	0.88 (0.48–1.61)	0.93 (0.37–2.32)	0.34 (0.08–1.47)	0.87 (0.30–2.51)	1.51 (0.56–4.07)	1.05 (0.30–3.61)	1.97 (0.88–4.40)	1.06 (0.49–2.28)
Malignant tumor	1.26 (0.57–2.76)	1.35 (0.45–4.12)	*3.14 (1.21–8.16)	–	0.49 (0.06–3.78)	–	1.09 (0.32–3.79)	1.13 (0.41–3.10)
Respiratory disease	0.96 (0.72–1.26)	0.89 (0.59–1.34)	1.26 (0.87–1.81)	1.04 (0.69–1.57)	0.85 (0.47–1.56)	0.88 (0.56–1.37)	0.95 (0.61–1.48)	1.01 (0.68–1.50)
Hay fever	*0.87 (0.757–0.999)	1.24	1.05	0.98	0.89	*1.28	1.17	1.00

		(1.00–1.54)	(0.86–1.27)	(0.79–1.21)	(0.65–1.22)	(1.03–1.60)	(0.93–1.46)	(0.82–1.23)
Mental illness								
Depression	1.01 (0.72–1.42)	*1.63 (1.06–2.50)	1.11 (0.68–1.82)	0.73 (0.41–1.28)	1.48 (0.82–2.68)	0.90 (0.50–1.59)	1.58 (0.99–2.53)	0.64 (0.38–1.09)
Schizophrenia	1.41 (0.66–2.98)	1.09 (0.40–2.98)	0.41 (0.10–1.78)	1.38 (0.47–4.06)	1.26 (0.28–5.56)	1.20 (0.40–3.61)	0.26 (0.04–1.96)	1.19 (0.40–3.49)
Other mental disease	0.99 (0.72–1.38)	1.18 (0.77–1.81)	0.67 (0.41–1.11)	1.28 (0.82–2.02)	1.01 (0.53–1.92)	0.93 (0.57–1.52)	0.99 (0.60–1.62)	1.20 (0.77–1.87)
Ophthalmic surgery								

Cataract surgery	1.64 (0.66–4.05)	0.76 (0.16–3.58)	0.80 (0.10–6.22)	0.78 (0.17–3.53)	0.39 (0.05–3.14)	–	0.55 (0.07–4.23)	0.92 (0.29–2.97)
LASIK	1.01 (0.59–1.75)	0.58 (0.20–1.67)	0.89 (0.37–2.14)	1.02 (0.42–2.48)	0.39 (0.05–3.14)	1.07 (0.40–2.83)	0.95 (0.37–2.47)	0.80 (0.33–1.95)
Other ophthalmic surgery	1.08 (0.72–1.63)	*1.89 (1.12–3.18)	0.77 (0.40–1.47)	**2.03 (1.20–3.43)	0.69 (0.23–1.94)	0.62 (0.26–1.44)	1.08 (0.55–2.12)	0.73 (0.38–1.41)
Lifestyle habits								
Coffee (every 1 cup per day increase)	0.96 (0.91–1.01)	1.04 (0.97–1.13)	1.03 (0.95–1.11)	0.97 (0.89–1.05)	1.06 (0.96–1.18)	1.06 0.97–1.16)	1.02 (0.93–1.11)	1.02 (0.95–1.10)
Contact lens use								

No	1 (reference)	1 (reference)	1 (reference)	1 (reference)	1 (reference)	1 (reference)	1 (reference)	1 (reference)
Current use	0.90 (0.77–1.05)	1.22 (0.96–1.56)	**1.41 (1.14–1.75)	1.10 (0.87–1.39)	**0.59 (0.40–0.88)	**1.58 (1.24–2.02)	0.84 (0.66–1.08)	*0.76 (0.61–0.96)
Past use	1.07 (0.85–1.33)	1.17 (0.83–1.66)	*1.42 (1.02–1.97)	0.85 (0.59–1.23)	1.35 (0.89–2.06)	*1.66 (1.13–2.43)	0.81 (0.56–1.19)	***0.52 (0.36–0.75)
Screen exposure (hours)*								
<4	1 (reference)	1 (reference)	1 (reference)	1 (reference)	1 (reference)	1 (reference)	1 (reference)	1 (reference)
4–8	0.92 (0.76–1.11)	0.93 (0.68–1.27)	1.07 (0.82–1.41)	1.02 (0.75–1.38)	0.85 (0.55–1.30)	0.97 (0.71–1.32)	1.28 (0.92–1.77)	0.99 (0.75–1.30)

>8	0.89 (0.72–1.10)	1.19 (0.85–1.66)	1.10 (0.82–1.49)	1.24 (0.90–1.73)	0.91 (0.56–1.46)	0.87 (0.62–1.23)	1.04 (0.72–1.50)	1.12 (0.83–1.53)
Periodic exercise (Yes *vs*. No)	0.96 (0.83–1.11)	0.92 (0.74–1.16)	1.22 (0.99–1.51)	0.94 (0.75–1.17)	**0.60 (0.43–0.82)	0.94 (0.74–1.18)	1.05 (0.83–1.33)	1.16 (0.93–1.43)
Sleeping time (every 1 hour per day increase)								
<6	**1.26 (1.08–1.48)	**1.45 (1.14–1.84)	0.98 (0.78–1.23)	1.13 (0.89–1.45)	0.90 (0.62–1.31)	0.95 (0.74–1.23)	0.80 (0.61–1.04)	*0.72 (0.57–0.91)
6–9	1 (reference)	1 (reference)	1 (reference)	1 (reference)	1 (reference)	1 (reference)	1 (reference)	1 (reference)
>9	1.10	1.17	0.97	*1.35	1.14	0.75	0.95	0.79

	(0.91–1.33)	(0.87–1.58)	(0.74–1.26)	(1.03–1.79)	(0.74–1.74)	(0.55–1.03)	(0.70–1.28)	(0.59–1.05)
Smoking (Yes *vs*. No)	0.89 (0.75–1.05)	*1.38 (1.08–1.77)	1.05 (0.82–1.33)	0.83 (0.64–1.08)	1.29 (0.91–1.81)	**1.44 (1.10–1.89)	1.09 (0.84–1.42)	0.88 (0.70–1.12)
Water intake (every 100 mL per day)	1.01 (0.99–1.02)	1.00 (0.98–1.03)	0.99 (0.97–1.02)	1.01 (0.99–1.03)	0.99 (0.96–1.03)	1.01 (0.99–1.04)	0.98 (0.96–1.01)	1.00 (0.98–1.02)
SDS >40	0.64 (0.55–0.75)	***1.77 (1.35–2.32)	1.00 (0.80–1.25)	1.13 (0.89–1.44)	**1.97 (1.34–2.90)	*1.34 (1.03–1.75)	1.20 (0.93–1.54)	*1.28 (1.02–1.60)

DE: dry eye; LASIK: laser-assisted *in situ* keratomileusis; SDS: Zung Self-rating Depression Scale

*$P < 0.05$, **$P < 0.001$, ***$P < 0.001$.

Supplementary Table 6. Survey questions.

Question	Variables	Variable details
	Age	Integer input, years
	Sex	Choose one {"Male," "Female"}
	Height	Integer input, cm
	Weight	Integer input, kg
	Race	Choose one {"American Indian or Alaskan Native," "Arabic," "Asian," "Black or African American," "Caucasian," "Hispanic, Latino, or Spanish," "Native Hawaiian or other Pacific Islander," "Other Race"}
Have you ever been diagnosed with hypertension?	Hypertension	Choose one {"No," "I am being treated for hypertension," "I have untreated hypertension," "I do not know"}
Have you ever been diagnosed with diabetes?	Diabetes (HbA1c)	Choose one {"Yes," "No"}. If "Yes," scale bar input of HbA1c (5–15)

Have you suffered from any of the following illnesses?	Systemic diseases	Multiple choice among {"Brain disease," "Blood disease," "Collagen disease," "Heart disease," "Kidney disease," "Liver disease," "Malignant tumor," "Respiratory disease," "N/A"}
Do you have hay fever?	Hay fever	Choose one {"Yes," "No"}
Do you suffer from the following mental illnesses?	Mental illness	Multiple choice among {"Depression," "Schizophrenia," "Other mental illness," "N/A"}
Have you ever been diagnosed as having dry eyes?	Past diagnosis of dry eye disease	Choose one {"Yes," "No"}
Have you ever undergone the following ophthalmic surgeries?	Ophthalmic surgery	Multiple choice among {"Cataract surgery," "LASIK," "Other ophthalmic surgery," "N/A"}
Have you used eye drops?	Eye drop	Choose one {"Yes," "No"}
How much coffee did you drink today?	Coffee intake	Scale bar input (0–10), cups

Have you ever used contact lenses?	Contact lenses	Choose one {"I have been using contact lenses," "I have used contact lenses in the past," "I have not used contact lenses"}
What type of contact lens have you been using (or did you use?)	Contact lens type	Multiple choice among {"Soft contact lens/daily," "Soft contact lens/bi-weekly," "Soft contact lens/monthly," "Soft contact lens/conventional contact lenses," "Hard contact lens," "Color contact lens/daily," "Color contact lens/bi-weekly," "Color contact lens/monthly," "Color contact lens/conventional contact lenses"}
How long have you been using contact lenses	Contact lens term	Scale bar input (0 to more than 10), year
How long do you wear contact lenses a day?	Contact lens duration per day	Scale bar input (0–24), hours
Please tell me how long you were exposed to the screen	Screen exposure time	Scale bar input (0–24), hours

today (Smartphone, PC, TV, etc.).		
How many hours do you exercise per week?	Periodic exercise	Scale bar input (0 to more than 10), hours
Please indicate at what time you went to bed last night.	Bedtime	Input drum bar, hours and minutes
Please indicate at what time you woke up this morning.	Wake-up time	Input drum bar, hours and minutes
Do you currently smoke, or have you smoked in the past?	Smoking	Choose one {"Yes," "No"}
How much fluid did you drink today?	Water intake	Scale bar input (0–2,000), mL
Daily subjective symptom questions		
Please answer each question using a 10-point scale	Stress level	Scale bar input (0, not at all to 10, I feel very stressed)
	Headache	Scale bar input (0, not at all to 10, very painful)

	Eye itching	Scale bar input (0, not at all to 10, very itchy)
Do you have any of the following symptoms that you aware of?	Concomitant symptoms	Multiple choice from {"Asthenopia," "Stiffness and pain of body axis muscle," "Mental fatigue," "N/A"}

HbA1c: hemoglobin A1c; LASIK: laser-assisted *in situ* keratomileusis; N/A: not applicable; PC: personal computer; TV: television.

Supplementary Table 7. Characteristics of each item of the J-OSDI.

	Non-symptomatic dry eye	Symptomatic dry eye						
J-OSDI questionnaire	Cluster 0	Cluster 1	Cluster 2	Cluster 3	Cluster 4	Cluster 5	Cluster 6	Cluster 7
Items, 0–4, median [IQR]	n=974 (27.1)	n=385 (10.7)	n=492 (13.7)	n=393 (10.9)	n=171 (4.8)	n=376 (10.5)	n=347 (9.7)	n=455 (12.7)
1. Eyes that are sensitive to light?	1 [0–1]	2 [1–3]	1 [1–2]	1 [1–1]	1 [1–3]	2 [1–3]	3 [3–4]	1 [1–1]
2. Eyes that feel gritty?	0 [0–1]	2 [1–3]	1 [1–2]	1 [1–2]	1 [1–1]	2 [1–3]	1 [1–2]	1 [0–1]
3. Painful or sore eyes?	0 [0–1]	2 [1–3]	1 [0–1]	1 [1–1]	1 [0–1]	1 [1–2]	1 [0–1]	1 [0–1]
4. Blurred vision?	0 [0–1]	3 [2–3]	1 [0–1]	1 [1–1]	2 [1–3]	1 [1–2]	1 [1–1]	1 [1–2]

5. Poor vision?	0 [0–1]	3 [2–3]	1 [0–1]	1 [0–1]	2 [1–3]	1 [1–2]	1 [0–1]	2 [1–2]
Vision-related function								
6. Reading?	0 [0–0]	2 [1–3]	0 [0–1]	0 [0–1]	1 [1–3]	1 [0–1]	1 [0–1]	1 [1–2]
7. Driving at night?	0 [0–0]	2 [1–3]	0 [0–1]	0 [0–0]	3 [2–3]	0 [0–1]	0 [0–1]	0 [0–1]
8. Working with a computer or bank machine (ATM)?	0 [0–0]	2 [1–3]	0 [0–1]	0 [0–0]	1 [0–2]	0 [0–1]	0 [0–1]	1 [0–1]
9. Watching TV?	0 [0–0]	2 [1–3]	1 [0–1]	0 [0–0]	1 [0–2]	1 [0–1]	0 [0–1]	1 [0–1]
Environmental triggers								
10. Windy conditions?	0 [0–0]	3 [1–3]	1 [1–2]	1 [0–1]	0 [0–1]	3 [3–4]	1 [0–1]	0 [0–1]

11. Places or areas with low humidity (very dry)?	0 [0–1]	3 [2–4]	2 [2–3]	1 [1–1]	1 [0–1]	3 [3–4]	1 [0–1]	1 [0–1]
12. Areas that are air-conditioned?	0 [0–0]	3 [2–4]	2 [1–3]	1 [0–1]	1 [0–1]	3 [2–4]	1 [0–1]	1 [0–1]

ATM: automated teller machine; IQR: interquartile range; J–OSDI: Japanese version of the ocular surface disease index.

Supplementary Table 8. Characteristics of each item of the SDS.

	Non–symptomatic dry eye	Symptomatic dry eye						
SDS questionnaire	Cluster 0	Cluster 1	Cluster 2	Cluster 3	Cluster 4	Cluster 5	Cluster 6	Cluster 7
Items, 0–4, median [IQR]	n=974 (27.1)	n=385 (10.7)	n=492 (13.7)	n=393 (10.9)	n=171 (4.8)	n=376 (10.5)	n=347 (9.7)	n=455 (12.7)
Ocular symptoms								
1. I feel down-hearted and blue	2 [1–2]	2 [1–3]	2 [1–2]	2 [1–2]	2 [1–2]	2 [1–2]	2 [1–2]	2 [1–2]
2. Morning is when I feel the best	3 [3–4]	4 [3–4]	3 [3–4]	3 [3–4]	3 [3–4]	4 [3–4]	3 [3–4]	3 [3–4]
3. I have crying spells or feel like it	1 [1–2]	2 [1–3]	2 [1–2]	2 [1–2]	2 [1–2]	2 [1–1]	2 [1–2]	2 [1–2]

4. I have trouble sleeping at night	1 [1–2]	2 [1–3]	2 [1–3]	2 [1–2]	2 [1–3]	2 [1–3]	2 [1–3]	2 [1–3]
5. I eat as much as I used to	1 [1–2]	2 [1–3]	1 [1–2]	1 [1–2]	21 [1–2]	1 [1–2]	1 [1–2]	1 [1–2]
6. I still enjoy sex	2 [1–3]	2 [1–3]	2 [1–3]	2 [1–3]	2 [1–3]	2 [1–3]	2 [1–3]	2 [1–3]
7. I notice that I am losing weight	1 [1–2]	1 [1–2]	1 [1–2]	1 [1–2]	1 [1–2]	1 [1–2]	1 [1–2]	1 [1–2]
8. I have trouble with constipation	1 [1–2]	2 [1–2]	1 [1–2]	1 [1–2]	2 [1–2]	2 [1–2]	2 [1–2]	1 [1–2]
9. My heart beats faster than usual	1 [1–1]	2 [1–2]	1 [1–2]	1 [1–2]	1 [1–2]	1 [1–2]	1 [1–2]	1 [1–2]
10. I get tired for no reason	2 [2–3]	3 [2–4]	3 [2–4]	3 [2–4]	3 [2–4]	3 [2–4]	3 [2–4]	3 [2–4]
11. My mind is as clear as it used to be	3 [2–3]	3 [3–4]	3 [2–3]	3 [2–4]	3 [2–4]	3 [2–4]	3 [2–4]	3 [2–4]

12. I find it easy to do the things I used to	2 [1–3]	2 [2–3]	2 [1–3]	2 [2–3]	2 [2–3]	2 [1–3]	2 [1–3]	2 [1–3]
13. I am restless and can't keep still	1 [1–2]	2 [1–3]	2 [1–2]	2 [1–2]	2 [1–3]	2 [1–3]	2 [1–3]	2 [1–2]
14. I feel hopeful about the future	3 [2–3]	3 [2–4]	3 [2–3]	3 [2–4]	3 [2–4]	3 [2–4]	3 [2–4]	3 [2–4]
15. I am more irritable than usual	2 [1–2]	2 [2–3]	2 [1–3]	2 [1–3]	2 [2–3]	2 [2–3]	2 [2–3]	2 [1–3]
16. I find it easy to make decisions	3 [2–3]	3 [2–3]	3 [2–3]	3 [2–3]	3 [2–3]	3 [2–3]	3 [2–3]	3 [2–3]
17. I feel that I am useful and needed	3 [2–3]	3 [2–4]	3 [2–4]	3 [2–3]	3 [2–4]	3 [2–4]	3 [2–4]	3 [2–3]
18. My life is pretty full	2 [2–3]	3 [2–3]	3 [2–3]	3 [2–3]	3 [2–4]	3 [2–3]	3 [2–3]	3 [2–3]

19. I feel that others would be better off if I were dead	1 [1–2]	1 [1–2]	1 [1–2]	1 [1–2]	1 [1–2]	1 [1–2]	1 [1–2]	1 [1–2]
20. I still enjoy the things I used to do	3 [2–3]	3 [2–4]	3 [2–3]	3 [2–3]	3 [2–4]	3 [2–4]	3 [2–3]	3 [2–3]

IQR: interquartile range; SDS: Zung Self-rating Depression Scale.
